# Multiple functional therapeutic effects of *Tn*P: A small stable synthetic peptide derived from fish venom in a mouse model of multiple sclerosis

**DOI:** 10.1371/journal.pone.0171796

**Published:** 2017-02-24

**Authors:** Evilin Naname Komegae, Tais Aparecida Matozo Souza, Lidiane Zito Grund, Carla Lima, Monica Lopes-Ferreira

**Affiliations:** Immunoregulation Unit, Special Laboratory of Applied Toxinology, Butantan Institute, São Paulo, Brazil; Centre National de la Recherche Scientifique, FRANCE

## Abstract

The pathological condition of multiple sclerosis (MS) relies on innate and adaptive immunity. New types of agents that beneficially modify the course of MS, stopping the progression and repairing the damage appear promising. Here, we studied *Tn*P, a small stable synthetic peptide derived from fish venom in the control of inflammation and demyelination in experimental autoimmune encephalomyelitis as prophylactic treatment. *Tn*P decreased the number of the perivascular infiltrates in spinal cord, and the activity of MMP-9 by F4/80+ macrophages were decreased after different regimen treatments. *Tn*P reduces in the central nervous system the infiltration of IFN-γ-producing Th1 and IL-17A-producing Th17 cells. Also, treatment with therapeutic *Tn*P promotes the emergence of functional Treg in the central nervous system entirely dependent on IL-10. Therapeutic *Tn*P treatment accelerates the remyelination process in a cuprizone model of demyelination. These findings support the beneficial effects of *Tn*P and provides a new therapeutic opportunity for the treatment of MS.

## Introduction

Multiple sclerosis (MS) is a chronic, autoimmune disorder of the central nervous system (CNS) leading to demyelination and neuronal loss associated with progressive neurological disability, including balance and mobility impairments, weakness, reduced cardiovascular fitness, ataxia, fatigue, bladder dysfunction, spasticity, pain, cognitive deficits, and depression [1]. Histopathologically, presents large, multifocal demyelinated sclerotic plaques scattered throughout the CNS. MS is estimated to affect over 2.1 million people in worldwide, and the prevalence has also increased, inflicting immense costs, both personal and societal.

Based on the frequency of symptoms, MS is classified into 4 types [2]: relapsing-remitting MS (RRMS); secondary-progressive form (SPMS); primary-progressive (PPMS) and primary-progressive with relapses. RRMS is the most common type diagnosed (85%), patients usually have recent lesions and attacks associated with some neurological dysfunction followed by periods without symptoms, most of these patients later have an evolution to the SPMS form where the remissions cease, and clinical symptoms deteriorate [3]. These patients have intense demyelination with the appearance of new lesions and with old lesions with intense glial cells death. The exact etiology of MS in not yet fully elucidated, but is generally believed to involve a combination of genetic [4] and environmental factors [5; 6] that lead to the development of CNS autoimmunity and progression of disease in susceptible individuals.

The pathological condition of MS relies on innate and adaptive immunity, where T lymphocytes recognize CNS antigens in dendritic cells (DCs) within the cervical lymph nodes that drain the cerebrospinal fluid. The super-expression of adhesion molecules and matrix metalloproteinases (MMP-2 and MMP-9) production enable Th1 and Th17 cells [7; 8; 9] to cross the blood–brain barrier (BBB). Experimental autoimmune encephalomyelitis (EAE) is a well characterized mouse model for MS. It is induced by immunization with myelin antigens such as myelin oligodendrocyte glycoprotein (MOG) in adjuvant or by adoptive transfer of myelin-specific T cells, resulting in inflammatory infiltrates and demyelination in the CNS and consequently axonal pathology resembling MS [10]. Besides these, toxin-induced demyelinating models like the cuprizone (bis-cyclohexanone-oxalyldihydrazone) model, is often used to investigate the molecular factors contributing to de- and remyelination [11; 12]. Liñares et al [13] suggest that oxidative/nitrative stress causes mitochondrial impairment and neuronal NOS (nNOS) is involved in cuprizone-induced demyelination.

With recent regulatory approvals, 10 disease-modifying therapies (DMTs) are available in many countries for RRMS [14; 15]. Management of the disease therefore solely aims to minimize symptoms, maintaining patients relapsing free, with no new lesion on magnetic resonance imaging (MRI) and no increase in expanded disability scale score (EDSS). DMTs have mostly failed as treatments for progressive multiple sclerosis, and there is a robust pipeline of experimental treatments at various stages of clinical development and so far the results of clinical trials have generally been disappointing. In this direction, new types of agents that beneficially modify the course of MS, stopping the progression and repairing the damage appear promising. Relevant phase 3 trial data recently presented the effectiveness of the Ocrelizumab, a humanized monoclonal antibody that selectively depletes CD20-expressing B cells, in the primary progressive form of the disease [16].

Recently, we identified new molecules denominated *Tn*P family derived from venom of *Thalassophryne nattereri* Brazilian fish, which has been utilized for drug discovery and development. The *Tn*P family was subjected to a patent application in several countries and currently is patented in the following: Europe (EP2046815B1); Mexico (MX300187); United States (US8304382B2); Canada (CA2657338C); China (CN101511861B); Hong Kong (HK1135406); India (IN256624); South Korea (KR1399175B1) and Japan (JP5635771B2). In Brazil, the invention is a pending patent application (BRPI0703175A2, date of filing: 20070719). The *Tn*P family invention refers to synthetic peptides with anti-inflammatory and anti-allergic activities containing a sequence of 13 L-amino acids in their primary structure. The structurally unique *Tn*P (C_63_H_114_N_22_O_13_S_4_, H-Ile-Pro-Arg-Cys-Arg-Lys-Met-Pro-Gly-Val-Lys-Met-Cys-NH2 with disulfide bond between Cys4 and Cys13 with 1514,8 Da), is a preclinical development candidate with a strong dossier.

In the present study, we employed myelin-dependent EAE model to clarify the anti-inflammatory effect and therapeutic potential of *Tn*P in MS and its potential to induces remyelination using the toxic model of demyelination induced by cuprizone. We found that *Tn*P therapeutic treatment successfully ameliorates EAE in an IL-10-dependent manner, inducing reduction of disease severity and delaying the onset of maximal symptoms. EAE mice treated with three different regimens of subcutaneous administration of *Tn*P have controlled the infiltration of leukocytes and inhibited the demyelination. The expansion of microglia and the activity of MMP-9 by F4/80+ macrophages were decreased after different regimen treatments. *Tn*P modulates the encephalitogenic CD4+ T cells, reducing in the CNS-infiltrating IFN-γ-producing Th1 and IL-17A-producing Th17 cells. Also, *Tn*P blocks the production of inflammatory cytokines in spleen and promotes the emergence of functional Treg not only in spleen, but also in the CNS. *Tn*P leads to accelerated remyelination in a cuprizone model of demyelination. The results of this study suggest that *Tn*P is a very active anti-inflammatory and pro-remyelinating new peptide which could be important for the treatment of demyelinating conditions as MS.

## Materials and methods

### Mice

Six to eight-week-old female (EAE model) or 8–10 week-old male (cuprizone model) C57BL/6 wild type (*WT*) mice weighting 16 to 18 g were obtained from a colony at the Butantan Institute, São Paulo, Brazil. Female knockout (*KO*) for IL-10 was obtained from a colony at Institute of Biomedical Sciences II, University of São Paulo, São Paulo, Brazil. Mice was kept in the same SPF animal unit maintained in sterile micro-isolators with sterile rodent feed and acidified water and housed in positive-pressure air-conditioned units (25°C, 50% relative humidity) on a 12 h light/dark cycle. This study was carried out in strict accordance with the recommendations in the Guide for the Care and Use of Laboratory Animals of the Brazilian College of Animal Experimentation. The protocol was approved by the Committee on the Ethics of Animal Experiments of the Butantan Institute (Permit Number: 747/10) and of University of São Paulo (Permit Number: 74/89, book 2).

### *Tn*P and MOG synthetic peptides

*Tn*P was manufactured under the patent holder’s proprietary method (Laboratório Cristália Produtos Químicos Farmacêuticos LTDA). The analysis of amino acid sequences of was done by a MALDI-ToF/PRO instrument (G&E Healthcare—Sweden). The three-dimensional structure was constructed by homology modeling using as templates homologous proteins uncovered by Protein Data Bank screening, based on the structure of antitrypsin (PDB code: 1ATU; S1 Fig).

Myelin oligodendrocyte glycoprotein trifluoroacetate lyophilized powder (MOG)_35-55_ peptide (P14391301 with 2581,4 Da and 95,2%) was purchased from GenScript (order 20615, Piscataway NJ USA).

### Analysis of clinical signs of active EAE

EAE was induced according to Mendel et al. [17]. Briefly, six to eight-week-old female C57BL/6 *WT* or IL-10 *KO* mice (n = 15 per group) received a subcutaneous injection (s.c.) in the tail base of 300 μg of MOG_35–55_ per animal emulsified in 100 μl incomplete Freund’s adjuvant (IFC, 263910, Difco) containing 500 μg of *Mycobacterium tuberculosis* H37RA (231141, Difco) on day 0. Immediately thereafter and again 48 h later, mice received an intraperitoneal injection (i.p.) of 500 ng of *Pertussis* toxin (P7208, Sigma-Aldrich, St Louis, MO, USA) diluted in 200 μl of sterile 0.9% saline. EAE progression was monitored for 30 d after immunization with MOG. Clinical sign scores of EAE were daily assigned as follows: 1, tail limpness; 2, impaired righting reflex; 3, hind limb paralysis; 4, hind- and forelimb paralysis; 5, death. The mean of monthly scores was calculated (S2 Fig). Mice was weight every day. All behavioral measurements were done in awake, unrestrained, age matched female mice. All tests were performed in an appropriate quiet room between 10 am and 4 pm. If necessary, food was provided on the cage floor. Prior injection of *Pertussis toxin* mice were anaesthetized with isoflurane. A humane endpoint was fixed using specific parameters as follow: EAE-mice consistently scored higher (≥4, complete hind limb paralysis or quadriparesis, and weight loss greater than 30%) were removed from the study and killed.

### Administration of *Tn*P

For treatment, mice (n = 15/group) was s.c. injected with 100 μl of *Tn*P at doses of 0.2; 0.4; 0.8; 1.5 or 3 mg/kg diluted in 0.9% saline. Mice was injected with *Tn*P every day from day 0 to 9 (Prophylactic treatment—during induction phase), from day 10 to 19 (Therapeutic treatment—during effector phase) or from day 0 to 19 (Continuous treatment—during induction and effector phases). The EAE controls were injected with 0.9% saline alone (Vehicle) (S2 Fig).

### Cell preparation from spleen and CNS

Mice (n = 5/group) was killed and spleens were removed at 7 days pos-immunization for analyses during the induction phase. The brain and the spinal cord were excised from mice perfused transcardially with ice-cold phosphate buffered saline (PBS) at peak of disease (17) or in late phase (30). Single-cell suspensions of splenic tissue were prepared by digestion with 1 mg/ml of type II collagenase (Roche) and 500 U DNase I (Sigma-Aldrich) following by mechanical disruption in GentleMacs dissociator (Miltenyi). Erythrocytes in spleens were lysed with 0.14 M NH_4_Cl and 17 mM Tris-Cl (pH 7.4). Additionally, the brain and spinal cord cell suspensions were prepared and centrifuged at 200 *g* for 10 min and resuspended in 4 ml of 30% isotonic Percoll (P1644, Sigma) diluted in HBSS and overlaid by equal volumes of 37% and 70% isotonic Percoll. The gradient was centrifuged at 800 *g* for 20 min and leukocytes were harvested from the 37% - 70% interface, washed, and counted.

### *In vitro* cell re-stimulation and cytokine secretion determination

Protein expression of intracellular cytokines was assessed by FACS analysis. Single cell suspensions were prepared from the spleen by mechanical disruption by forcing the tissue through a nylon mesh with 70 μm pore size (Cell Strainer, BD), and the pellets were resuspended in PBS with 10% fetal calf serum (FCS). Cells were then stimulated with medium containing 50 ng/ml PMA (Sigma-Aldrich), 1 μg/ml ionomycin (Sigma-Aldrich), and 1 μl/ml monensin (GolgiStop; BD) at 37°C and 5% CO_2_ for 4 h. After staining of surface markers, cells were fixed and permeabilized (Cytofix/Cytoperm and Perm/Wash buffer; BD), followed by staining with monoclonal antibodies to mouse PerCP5.5-FoxP3 (45-5773-82, eBioscience), allophycocyanin-IL-4 (554436, BD Biosciences), allophycocyanin-IFN-γ (IC485A, R&D Systems); and FITC-IL17A (IC421F, R&D Systems). Cytokine secretion (IL-6, TNFα, MCP-1 or CCL2, IFN-γ, IL-12p70, and IL-10) was measured in supernatants collected from re-stimulated cells using a Mouse Inflammation Cytometric Bead Array (CBA 552364) according to the manufacturer’s instructions (BD Biosciences). Briefly, 50 μl of sample were mixed with 50 μl of the mixed capture beads and 50 μl of the mouse PE detection reagent. The tubes were incubated at room temperature for 2 h in the dark, followed by a wash step. The samples were then resuspended in 300 μl of wash buffer before acquisition on the FACSCalibur flow cytometer. The data were analyzed using the CBA software (BD Biosciences). Standard curves were generated for each cytokine using the mixed bead standard provided in the kit, and the concentration of cytokine in the supernatant was determined by interpolation from the appropriate standard curve (IL-6: 5 pg/ml, TNFα: 7.3 pg/ml, MCP-1: 52.7 pg/ml, IFN-γ: 2.5 pg/ml, IL-12p70: 10.7 pg/ml, and IL-10: 17.5 pg/ml).

### Flow cytometry analysis

Spleen, brain, and the spinal cord of EAE mice treated with *Tn*P or vehicle were harvested and cell suspensions were prepared for cytometer analysis. For surface staining, single-cell suspensions (1 x 10^6^ cells in 100 μl) were treated with 3% mouse serum of naive mice and then incubated for 30 min in ice with specific anti-mouse Abs fluorochromes-conjugated or purified Abs followed by secondary Abs fluorochromes-conjugated purchased from BD Biosciences, R&D Systems or eBioscience: FITC-CD11c (553801, BD Biosciences), PerCP-Cy5.5-CD11b (550993, BD Biosciences), PE-IA/IE (557000, BD Biosciences), PE-CD40 (553791, BD Biosciences), PE-CD80 (553769, BD Biosciences), PE-CD86 (553692, BD Biosciences), PerCP-Cy5.5-CD45R/B220 (15-0452-83, eBioscience), PE-CD274 (558091, BD Biosciences), PE-CD237 (557796, BD Biosciences), FITC-CD4 (553729, BD Biosciences), PE-CD4 (557308, BD Biosciences), PE-CD18 (553293, BD Biosciences), PE-CD154 (553658, BD Biosciences), PerCP-Cy5.5-CD69 (551113, BD Biosciences), PE-CD25 (553075, BD Biosciences), FITC-CD19 (557398 or 553785, BD Biosciences), PE-CD5 (553022, BD Biosciences), allophycocyanin-CD1d (17-0011-82, eBioscience), unlabeled rat anti-mouse CD45 (MAB114, R&D Systems) and anti rat Ig PerCP5.5 (F0115, R&D Systems) for 30 min on ice. Cells were washed three times in RPMI medium and re-suspended in paraformaldehyde 1% for the cytofluorometric analysis. Negative-controls were used to set the flow cytometer photomultiplier tube voltages, and single-color positive controls were used to adjust instrument compensation settings. Cells were examined for viability by flow cytometry using side/forward scatter characteristics or 7-AAD exclusion. Data (50,000 events acquired per sample) were acquired using a four-color FACSCalibur flow cytometer equipped with CellQuest software (Becton-Dickinson, San Jose, CA). Data were recorded as percent of fluorescent positive cells, MFI or absolute number per organ.

### Assessment of histological EAE

To evaluate the histological manifestations of EAE, mice (n = 5/group) was killed on day 17. The spinal cords were removed and fixed in buffered formalin 4%. Paraffin-embedded sections of spinal cord were stained with hematoxylin and eosin (H&E) or with Luxol fast blue (LFB) for analysis of inflammation or demyelination, respectively. Histopathological examination was performed in a blinded fashion. Counts of immune reactive cells (nucleated only, cell area ranging from 4 to 100 μm) in 1 mm^2^ area of cervical spinal cord were performed with an upright microscope (Axiolab, Carl Zeiss, Oberkochen, Germany) coupled to a photographic camera (AxioCam Icc1, Carl Zeiss, Oberkochen, Germany) using a 10/0.3 longitudinal distance objective/numeric operture and 1.6 optovar (Carl Zeiss, Oberkochen, Germany). Demyelination in the spinal cord was scored as: 0, none; 1, rare foci; 2, a few areas of demyelination; 3, large (confluent) areas of demyelination.

### Analysis of gelatin zymography on polyacrylamide gel

To evaluate the proteolytic activity of matrix metalloproteinases-9 (MMP-9) in EAE mice or under treatment (n = 5/group), the zymography test was performed on the homogenate of spinal cord at day 17. Crude spinal cord extracts were prepared by homogenization in ice-cold buffer (1 M NaH_2_PO_4_, 1 M sucrose, 0.5 M EDTA) with protease inhibitors (88665, Pierce); samples were centrifuged and the solubilized fraction was collected. Briefly, 20 μl of proteins were added to non-denaturing loading buffer and subjected to electrophoresis on Novex 10% Zymogram SDS-PAGE with 0.1% gelatin as substrates incorporated into the gel (EC61752 Box). After electrophoresis and washing twice with 2.5% (v/v) Triton X-100, the gels were incubated overnight at 37°C, and immersed in a developing buffer (50 mmol/l TRIS-hydrochloric acid, pH 7.4, supplemented with 5 mmol/l calcium chloride, 10–6 mol/l zinc chloride, and 0.02% sodium azide). Afterwards, the gels were stained with 0.25% Coomassie brilliant blue R-250 for 1 h, and de-stained to expose proteolytic bands in 50% methanol and 10% acetic acid for 1 h. Recombinant MMP-9 (Calbichem CO., San Diego, CA) was included as positive control of the proteinase activity bands. The proteinase activity was evidenced as clear bands (zones of gelatin degradation) against the blue background of stained gelatin. Gels were scanned and converted to grayscale in Adobe Photoshop. Band intensities were quantified by ImageJ software using the semi-automated Gel Analysis Tool. Results were expressed as densitometry units (DU).

### *In situ* zymography and immunofluorescence

To detect MMP activity produced by macrophages, we used *in situ* zymography to localize net gelatinolytic activity in F4/80 positive macrophages in spinal cord sections [18]. The assay is based on the increase of fluorescence of intramolecularly quenched fluorescein isothiocyanate-labeled DQ-gelatin on proteolytic cleavage. Frozen in Tissue-Tek O.C.T. Compound (4583, Sakura), non fixed 25 μm spinal cord sections were thawed and incubated for 1 h at 4°C in a humid chamber with 1/250 anti-mouse F4/80 antibody (377009, Santa Cruz). The sections were rinsed in PBS for 5 min tree fold and incubated for 1 h at 4°C in a humid, dark chamber with 1/200 anti-mouse Ig antibody Texas red conjugated (2979, Santa Cruz). After rinsed in PBS, the sections were incubated for 3 h at 37°C in a humid, dark chamber in reaction buffer containing 25 μg/ml of FITC-labeled DQ^TM^-gelatin (E-12055, EnzChek gelatinase/collagenase assay kit, Molecular Probes, Eugene, OR). The sections were rinsed in PBS for 10 min and fixed in 4% formaldehyde for 20 min then mounted in fluorescent mounting medium (VECTOR, Burlingame, CA). Tissue sections were imaged with an inverted fluorescence microscope Olympus IX81 with a saline immersion objective (SW40/0.75 numerical aperture, Zeiss, Jena, Germany) coupled with a photographic camera (AxioCam Icc1, Carl Zeiss, Oberkochen, Germany) using a Cell R program (Olympus, Hamburg, Germany) and AutoQuantX3 program for deconvolution.

### Induction of demyelination by cuprizone and *Tn*P treatment

Demyelination was induced by feeding 8–10 week old male C57BL/6 mice with a diet containing 0.2% (wt/wt) cuprizone (biscyclohexanone oxaldihydrazone, 14690. Sigma-Aldrich) mixed into a ground Breeder Chow 2000 (Purina, Richmond, IN) for up to 6 consecutive weeks as previously described [11]. The mice was daily monitored for clinical signs and killed at 6 weeks of diet to determine neuropathology and to conduct histological analyzes. After 6 weeks, healthy control or cuprizone mice were maintained on a normal diet for the duration of 6 weeks. For the therapeutic study, groups of at least five mice were s.c. injected with 100 μl of *Tn*P at dose of 3 mg/Kg for 3 alternate days per week and killed after 1, 2, 3, 4, 5 or 6 weeks of normal feeding. Clinical sign scores of neurological disorder were daily assigned as follows: 1, tail limpness; 2, impaired righting reflex; 3, hind limb paralysis; 4, hind- and forelimb paralysis; 5, death (S3 Fig).

### Histological and immunofluorescence evaluation in the *corpus callosum*

Mice was anesthetized and perfused through the heart with 0.1 M phosphate buffer followed by either 4% paraformaldehyde for paraffin embedding or immunofluorescence analyses. In each experiment, the brains of two cuprizone-treated mice at each time point were further post-fixed *in situ* overnight at 4°C in the same fixative and removed. The cerebrum was coronally sectioned to expose the corpus callosum. The paraffin embedded tissues were sectioned at 7 μm in thickness, and the sections were stained with hematoxylin and eosin (H&E) and Luxol fast blue (LFB) stains. For immunofluorescence analyses, the tissue samples were embedded in O.C.T., cut into 10 μm transverse sections on a cryotome and mounted on glass slides. The slides were first immersed in a solution containing 1% NaOH in 80% ethanol for 5 min. They were rinsed for 2 min in 70% ethanol and for 2 min in distilled water, then incubated in 0.06% potassium permanganate solution for 10 min. Following a water rinse for 2 min, slides were transferred to the Fluoro-Jade C staining solution and stained for 10 min. The proper dilution was accomplished by first making a 0.01% stock solution of Fluoro-Jade C dye (AG325, Millipore) in distilled water and then adding 1 ml of the stock solution to 99 ml of 0.1% acetic acid. Slides were washed three times each for 1 min and then air-dried on a slide warmer at 50°C for 30 min. Cell nuclei was visualized after DAPI—4',6-diamidino-2-phenylindole (sc-300415, Santa Cruz) incubation for 10 min at room temperature. Fluoromount-G (00–4958, eBioscience) was added to the slides prior to mounting with cover slips. Tissue sections were imaged with an inverted fluorescence microscope Olympus IX81 with a saline immersion objective (SW40/0.75 numerical aperture, Zeiss, Jena, Germany) coupled with a photographic camera (AxioCam Icc1, Carl Zeiss, Oberkochen, Germany) using a Cell R program (Olympus, Hamburg, Germany) and AutoQuantX3 program for deconvolution. The fluorescein/FITC filter system was used for visualizing Fluoro-Jade C staining and images were captured for demonstration.

### Statistical analysis

All values were expressed as mean ± SEM of one experiment representative of 2–3 experiments. Parametric data were evaluated using an analysis of variance, followed by the Bonferroni test. Non-parametric data were assessed using the Mann–Whitney test. Differences were considered statistically significant at *p* < 0.05. The GraphPad Prisma 6 statistical package was employed.

## Results

### *Tn*P treatment ameliorates EAE in an IL-10-dependent manner

Initially to investigate the direct effect of *Tn*P on the pathogenesis of EAE, Bl6 *WT* female mice was actively induced with MOG_35–55_ and prophylactic treated with several doses of s.c. injection of *Tn*P. Our results in Supporting Information showed that *Tn*P at 0.4 mg/Kg delayed the onset of signs of EAE (**S4*A* Fig**) after MOG_35–55_ inoculation, prevented the maximal clinical signs of EAE (**S4*B* Fig**), demyelination (**S4*C* Fig**), and MMP-9 activity (**S5*A* Fig**). The higher dose of *Tn*P, 3 mg/Kg showed to be more efficient to reduce the maximal score of disease and demyelination. No differences between *Tn*P at 0.2 mg/Kg and control EAE were observed. Treatment with *Tn*P, in turn, significantly decreased the number of the perivascular infiltrates found in the analyzed sections of spinal cord (**S5*B* Fig**) from the dose of 0.8 mg/Kg, and an increment of the weight of mice during the effector phase of EAE was induced by *Tn*P from the dose of 0.2 mg/Kg (**S5*C* Fig**).

**Fig 1:**
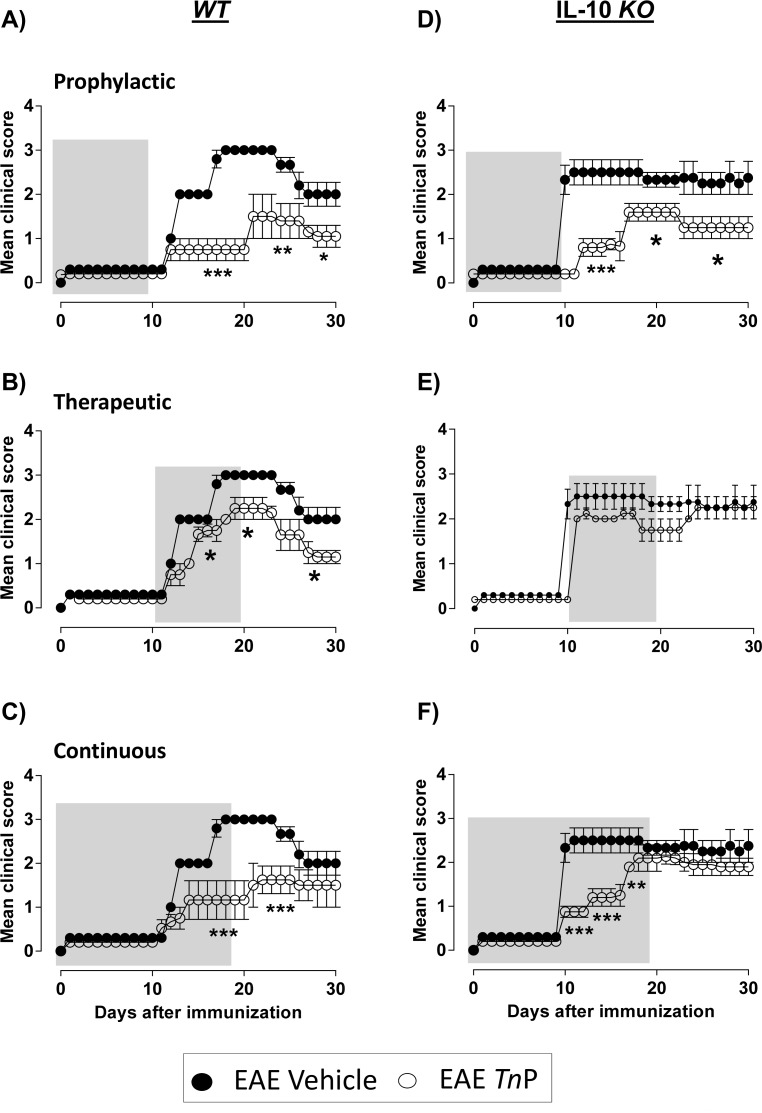
*Tn*P treatment ameliorates EAE in an IL-10-dependent manner. C57BL/6 *WT* or IL-10 *KO* mice (n = 15/group) immunized with (MOG)_35-55_ peptide in incomplete Freund’s adjuvant added with *M*. *tuberculosis* were injected 2 times with *Pertussis* toxin after immunization. Mice was scored (0–5) daily for 30 d for evidence of clinical disease (n = 15/group). Mice was treated with 3 mg/kg of *Tn*P diluted in 0.9% saline every other day starting at the day of immunization as following: day 0 to 9 (Prophylactic—***A***, ***D***), from day 10 to 19 (Therapeutic—***B***, ***E***) or from day 0 to 19 (Continuous—***C***, ***F***). The EAE controls were injected with 0.9% saline alone (Vehicle). Data represent mean ± SEM. *p < 0.05 and **p < 0.01 and *** *p* < 0.001 compared with vehicle-treated EAE-mice.

Next, we chose *Tn*P at 3 mg/Kg to treat Bl6 *WT* or IL-10 *KO* mice induced to EAE. We observe that EAE was strongly induced with 100% incidence and reached a peak score of 3 at day 17 (black circles). The disease started with clinical score of 1 at day 12, reaching a score of 2 between days 13 to 16. Between days 17 to 23, maximal symptom score of 3 was observed. At days 24 and 25 the symptoms declined to score of2.5, remaining between days 26 to 30 at score of 2. Mean symptom severity during the course of the disease was score at 2.1 (**Fig 1*A*, 1*B*** and **1*C*** and **Table 1**).

**Table 1:** Clinical scores of different regimens of *Tn*P treatment.

Treatment	Disease Incidence	Day of Onset	Maximal Score	Mean Clinical Score
**Vehicle**	15/15	17 ± 0.10	3.0 ± 0.23	2.1 ± 0.21
***Tn*P Prophylactic**	3/15***	21 ± 0.22 **	1.5 ± 0.11***	1.2 ± 0.10***
***Tn*P Therapeutic**	3/15***	19 ± 0.20*	2.1 ± 0.16***	1.5 ± 0.13***
***Tn*P Continuous**	3/15***	21 ± 0.22**	1.5 ± 0.11***	1.1 ± 0.10***

After induction of EAE with MOG_35–55_, *WT* mice was s.c. treated with *Tn*P (3 mg/kg) from days 0 to 9 (Prophylactic), from days 10 to 19 (Therapeutic) or from days 0 to 19 (Continuous) and scored (0–5) daily during 30 days for evidence of clinical disease signs (n = 15/group). The controls were injected with 0.9% saline (vehicle). Data represent mean ± SEM.

*p < 0.05

**p < 0.01 and

*** *p* < 0.001 significant differences between *WT* vehicle- and *Tn*P-treated EAE mice.

Bl6 *WT* mice with EAE undergoing prophylactic s.c. treatment with 3 mg/Kg of *Tn*P (**Fig 1*A***, open circles) showed an improvement of the symptoms, presenting between days 11 to 20 clinical score of 1, which increased to 1.5 between days 21–26, and declined to score 1 between days 27 and 30. In these animals we observe that prophylactic *Tn*P treatment delayed the onset of appearance of maximal symptoms (from 17 to 21) and decreased the mean intensity of symptoms to 1.2 compared to 2.1 of vehicle-treated EAE mice (**Table 1**). Therapeutic *Tn*P treatment of Bl6 *WT* mice with EAE (**Fig 1*B***) also induced reduction of disease severity with clinical score of 1 between days 12 to 14, which increased to score of 1.7 between days 15 to 18, reaching a high intensity of symptoms with score of 2.1 between days 19 to 23. At days 24–26, the score decreased to 1.7 reaching score of 1.2 between days 27–30. In therapeutic treated mice it was observed a delay in the onset of maximal symptoms (from 17 to 19) and the mean intensity of symptoms was 1.5 compared to 2.1 of vehicle-treated EAE mice (**Table 1**). The continuous s.c. treatment with *Tn*P (**Fig 1*C***) reduced the clinical score to 0.5 between days 11 to 13, between days 14 to 20 the score symptom was 1.1; between days 21 to 25 was 1.5 and declined to 1.3 between days 26–30. The continuous treatment with *Tn*P delayed the onset of maximal symptoms (from 17 to 21) and the mean intensity of symptoms to 1.1 compared to 2.1 of vehicle-treated EAE mice. All regimens of treatment with s.c. *Tn*P reduced the incidence of disease to 20% (**Table 1**).

IL-10 plays a more critical role in the regulation of EAE by regulating autopathogenic Th1 response [19]. In the **Fig 1*D***, **1*E*** and **1*F*** and **Table 2** we confirmed the higher susceptible to the induction of EAE of Bl6 IL-10 *KO* mice, with mean maximal score of 2.3 at day 10, which remained throughout the experiment. In the absence IL-10 the prophylactic treatment with *Tn*P (**Fig 1*D***) kept the symptoms at the score of 0.9 between days 12 to 16, which increased to score of 1.7 between days 17 to 22, and reaching score 1.1 between days 23 to 30. In this group of *KO* mice the prophylactic *Tn*P treatment reduced the mean intensity of score to 1.2 compared to 2.3 in vehicle-treated IL-10 *KO* EAE mice, and delayed the peak of onset of maximal symptoms from day 10 to day 17. IL-10 *KO* mice induced to EAE and treated therapeutically with *Tn*P (**Fig 1*E***) presented score of 2 between days 11 to 17, score of 1.9 between days 18 to 22, and score of 2.1 between days 23 to 30. In this group, although *Tn*P delayed the day of maximal symptoms (10 to 23), it did not control the symptoms, maintaining the mean clinical score of 2.0 compared to 2.3 in vehicle-treated IL-10 *KO* EAE mice. Compared to the *WT* EAE mice also therapeutically treated with *Tn*P, we found that the absence of IL-10 determined the maintenance of high values of the mean maximal score (2.1 compared to 2.3) and the mean intensity of symptoms (2 compared to 2.3), demonstrating a beneficial effect of IL-10 to *Tn*P effect (**Table 2**). IL-10 *KO* mice induced to EAE and treated continuously with *Tn*P (**Fig 1*F***) showed symptoms with score of 0.9 between days 10 to 12, score of 1.1 between days 13 to 16, score of 2.0 between days 17 to 23, and score of 1.9 between days 24 to 30. In this group of *KO* mice, *Tn*P decreased the mean clinical score to 1.5 compared to 2.3 of IL-10 *KO* mice induced to EAE without treatment, and delayed the peak onset of maximal symptoms from day 10 to day 17.

**Table 2:** Effect of IL-10 on clinical scores of *Tn*P-treated EAE mice.

Treatment	Disease Incidence	Day of Onset	Maximal Score	Mean Clinical Score
***WT* Vehicle**	15/15	17 ± 0.10	3.0 ± 0.23	2.1 ± 0.21
***WT* Therapeutic *Tn*P**	3/15	19 ± 0.20*	2.1 ± 0.16***	1.5 ± 0.13***
***IL-10 KO Vehicle***	15/15	10 ± 0.17	2.3 ± 0.10	2.3 ± 0.06
**IL-10 *KO* Therapeutic *Tn*P**	6/15	11 ± 0.07#	2.1 ± 0.09#	2.0 ± 0.08#

*WT* or IL-10 *KO* mice treated or not therapeutically wit *Tn*P were scored (0–5) daily during 30 days for evidence of clinical disease signs (n = 15/group). The controls were injected with 0.9% saline (vehicle). Data represent mean ± SEM.

*p < 0.05 and

*** *p* < 0.001 significant differences between *WT* vehicle- and *Tn*P-treated EAE mice

# *p* < 0.05 significant differences between *WT Tn*P-treated EAE mice and IL-10 *KO Tn*P-treated EAE mice.

### *Tn*P controls the infiltration of leukocytes and demyelination

**Fig 2:**
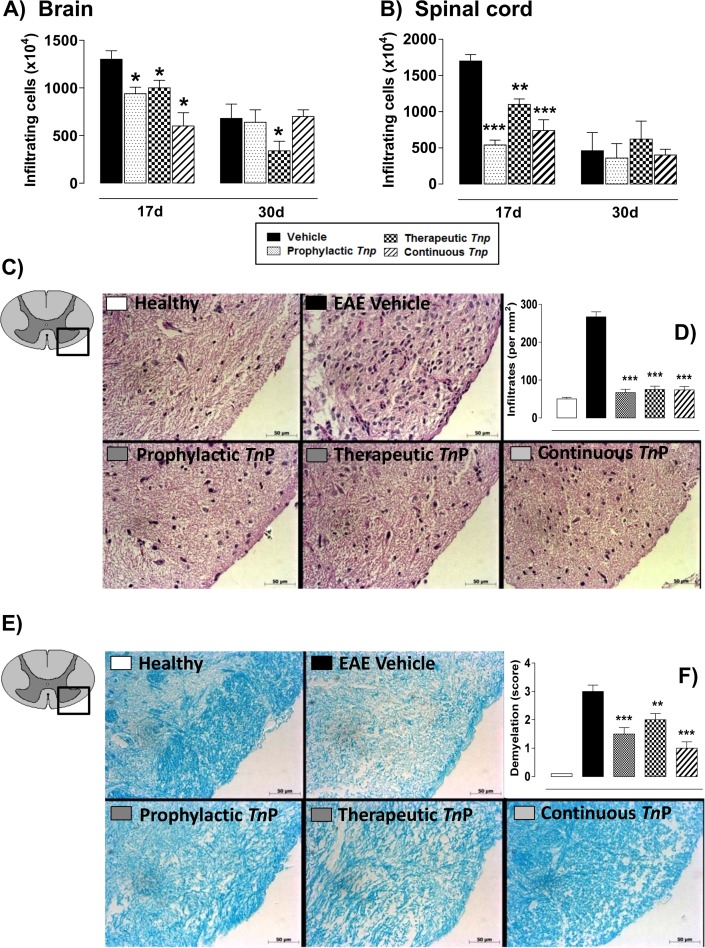
*Tn*P inhibits the infiltration of leukocytes to CNS and demyelination. Quantification of brain (***A***) and spinal cord (***B***) cellular infiltrates in pooled tissue homogenates collected at days 17 and 30 from vehicle- or *Tn*P-treated EAE mice (n = 5/group). Spinal cords from healthy and EAE mice treated with vehicle or *Tn*P were removed on the peak of disease (17) and stained in with H&E (***C***) in the upper panels or Luxol fast blue in the lower ones (***E***). The quantification of cells (***D***) and demyelination (***F***) were evaluated blindly. Representative sections are shown. Data represent mean ± SEM. *p < 0.05 and **p < 0.01 and *** *p* < 0.001 compared with vehicle-treated EAE-mice.

The infiltration of auto-reactive T cells and then macrophages into the CNS marks the onset of symptoms in EAE. Next, we evaluated whether the different regimens of *Tn*P treatment blocked the infiltration of leukocytes at days 17 and 30 in brains and spinal cords of *WT* EAE-mice. Our results in **Fig 2** show that at the peak of disease (17) the prophylactic, therapeutic or continuous treatments with *Tn*P decreased the cellular infiltrate in the brain (28%, 23% and 54%, respectively—**Fig 2*A***) and in the spinal cord (68%, 35% and 56%, respectively—**Fig 2*B***). Only the therapeutic treatment with *Tn*P sustained in the brain the decrease until day 30. In **Fig 2*C*** and **2*D***, the H&E stained sections of spine cords obtained at day 17 from EAE-mice showed inflammatory lesions with dense and focal mononuclear infiltrates compared to healthy mice. In contrast, there was a marked reduction of these lesions after *Tn*P treatment at different regimens. We also observe in **Fig 2*E*** and **2*F*** that all regimens of *Tn*P treatment suppressed the demyelination in the spinal cord of EAE-mice. The modulation of leukocyte influx and inhibition of demyelination induced by *Tn*P treatment were not reversed in IL-10 *KO* EAE *Tn*P-treated mice (**S6 Fig** and **S7 Fig**).

### *Tn*P decreases microglia and the activity of MMP-9 by F4/80+macrophages

**Fig 3:**
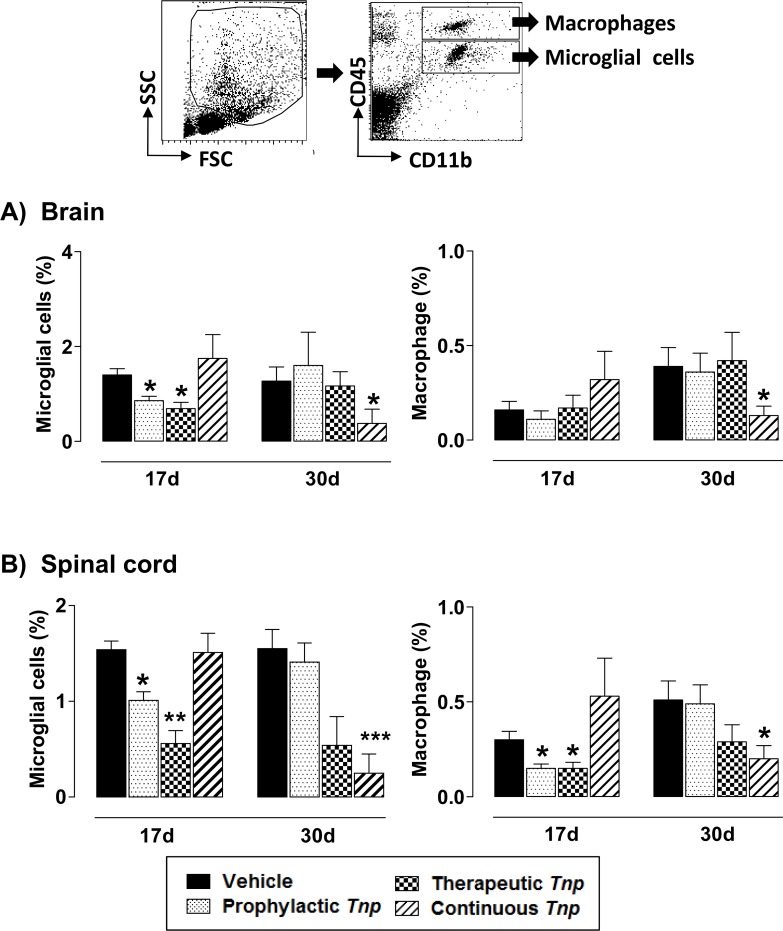
*Tn*P reduces the expansion of microglia and infiltration of macrophages in CNS. At days 17 and 30 post immunization, CNS-infiltrating leukocytes were isolated from pooled brain (***A***) and spinal cord (***B***) homogenates of EAE mice treated with vehicle or *Tn*P (n = 5/group), and the percentages of microglia (CD11b^low^CD45^low^) and infiltrating macrophages (CD11b^high^CD45^high^) as depicted in dot plot were analyzed by flow cytometry after acquisition of 50,000 events. Values in the bar graphs are the mean ± SEM. *p < 0.05 and **p < 0.01 and *** *p* < 0.001 compared with vehicle-treated EAE-mice.

Microglia and the presence of macrophages in the CNS cooperate to destruction of myelin barrier via induction of the release of inflammatory mediators such as free radicals, reactive oxygen intermediates, nitric oxide and MMP [20]. Next, in **Fig 3** we evaluated the percentage of microglia (CD11b^low^CD45^low^) and macrophages (CD11b^high^CD45^high^) at the peak of disease (17) or at chronic phase (30) in the brain (***A***) or spinal cord (***B***) of EAE-mice after all regimens of *Tn*P treatment. We showed that *Tn*P applied in the prophylactic or therapeutic regimens decreased the expansion of microglial cells at day 17, both in the brain (39% and 47%, respectively—**Fig 3*A***) and in the spinal cord (33% and 60%, respectively—**Fig 3*B***). We also observe a reduction in macrophage infiltration only in spinal cord in EAE-mice treated with prophylactic and therapeutic regimens of *Tn*P (50% and 49%, respectively). Then, we observe that only *Tn*P applied in continuous regimen maintained low the percentage of microglia and macrophages in brain (**Fig 3*A***) and in spinal cords (**Fig 3*B***) at chronic phase (30). The modulation induced by *Tn*P treatment was not reversed in IL-10 *KO* EAE *Tn*P-treated mice (**S8 Fig**).

**Fig 4:**
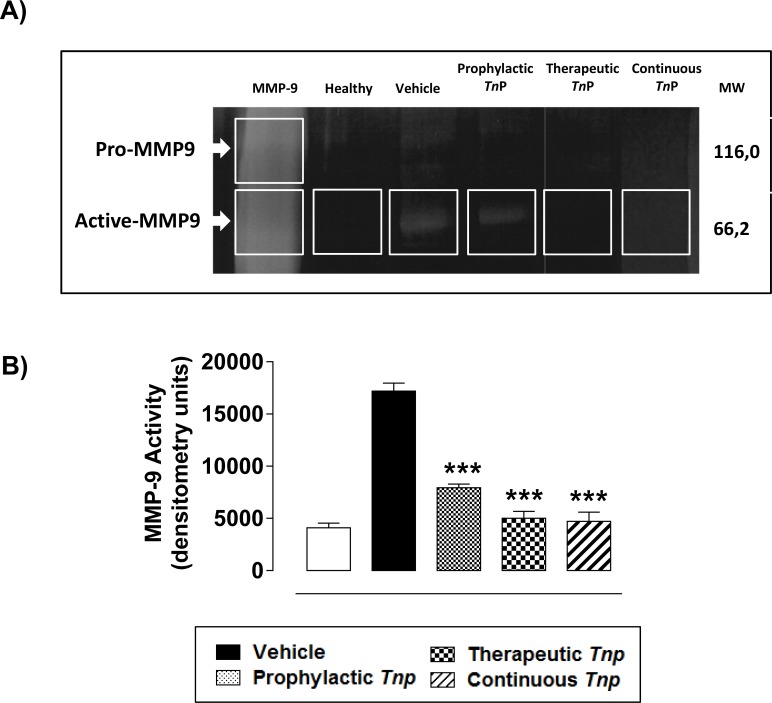
Gelatinase activity in EAE spinal cords is reduced by *Tn*P. Gelatin gel zymography of pooled tissue homogenates of spinal cords at day 17 from healthy and EAE mice treated with vehicle or *Tn*P (n = 5/group) shows active and pro-form of MMP-9 (***A***). Bar graphs (***B***) show densitometry quantification of representative gel zymography data. rMMP-9 was used as standard. Data represent mean ± SEM. ***p < 0.001 compared with vehicle-treated EAE-mice.

Our results depicted in **Fig 4** show that the prophylactic treatment with *Tn*P partially decreased the production of active MMP-9 as compared to the EAE vehicle treated mice, while therapeutic and continuous regimens maintained MMP-9 activity in low levels similar to healthy mice (**Fig 4*A*** and **4*B***). The modulation induced by *Tn*P treatment was not reversed in IL-10 *KO* EAE *Tn*P-treated mice (**S9 Fig**).

**Fig 5:**
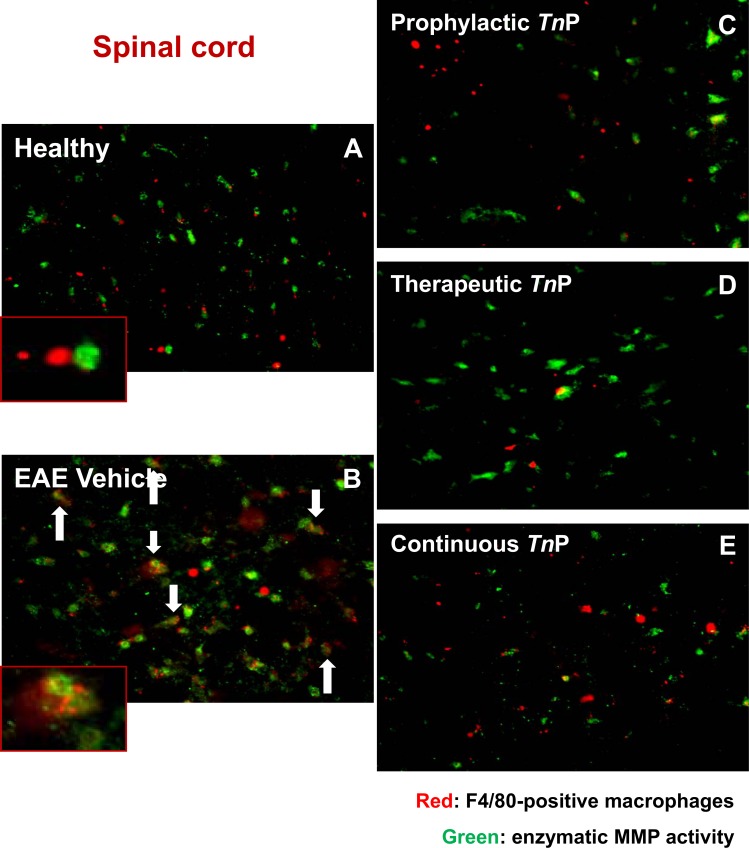
Macrophages are crucial for gelatinase activity. Immunofluorescence for F4/80-positive macrophages (red) in spinal cord sections of healthy (***A***) and EAE mice treated with vehicle (***B***) or *Tn*P (***C***-***E***) at day 17 (n = 5 mice/group) in slides incubated with DQ gelatin (FITC-labeled gelatin). Gelatinase activity was visualized by cleaving gelatin (green). Images are from healthy and EAE mice treated with vehicle or *Tn*P and representative sections are shown.

We next confirmed by *in situ* gelatin zymography that macrophages express gelatinolytic activity in the injured spinal cord of EAE vehicle treated mice (**Fig 5*B***) compared to healthy mice (**Fig 5*A***). Gelatinolytic activity in *WT* vehicle-treated EAE mice co-localized with F4/80+ macrophages at the lesion epicenter at day 17 (**Fig 5*B***). In contrast, the prophylactic (**Fig 5*C***), therapeutic (**Fig 5*D***) or continuous (**Fig 5*E***) regimens of treatment with *Tn*P reduced the distribution of gelatinolytic activity surrounding F4/80+ macrophages.

### *Tn*P treatment induces regulatory cells in spleen and in CNS and blocks the production of inflammatory cytokines

**Fig 6:**
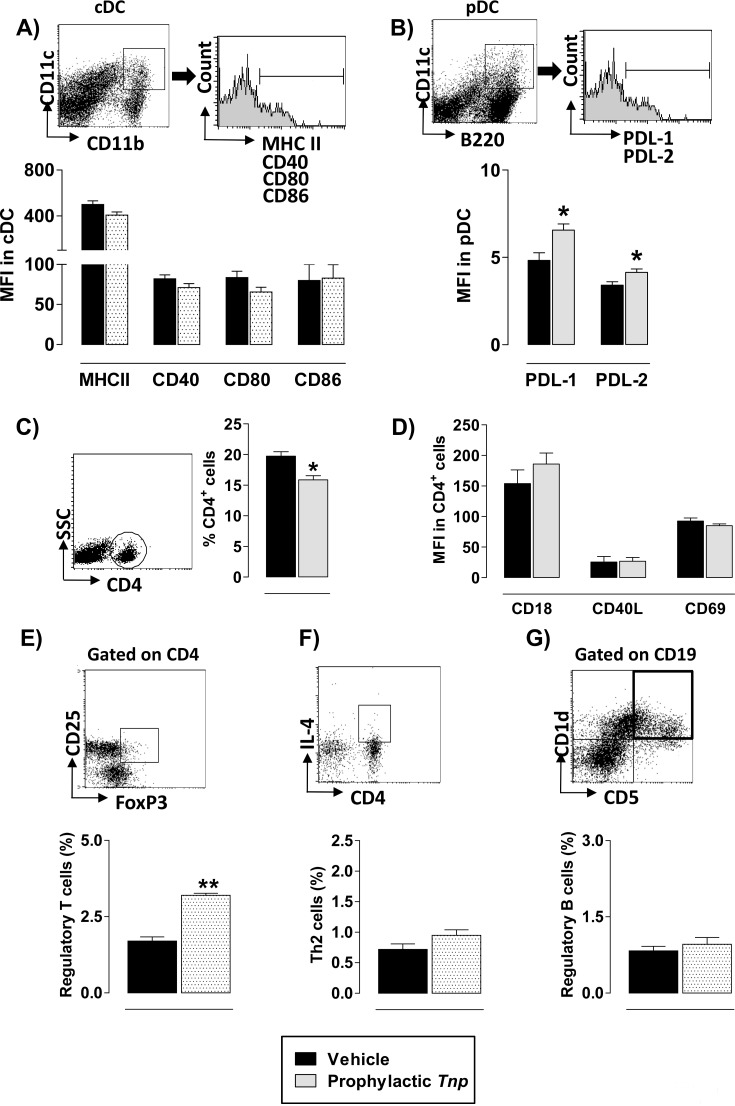
*Tn*P acts systemically during the induction phase modulating DCs and induces regulatory cells. Splenocytes from EAE mice treated with vehicle or *Tn*P (n = 5/group) were isolated at day 7, and analyzed. ***A***) The MFI of MHC class II, CD40, CD80, CD86 in cDC (CD11c+CD11b+) and ***B***) MFI of PDL-1 and PDL-2 in pDC (CD11c+B220^low^) were analyzed by flow cytometry (50,000 events). ***C***) The percentage of CD4+ cells, ***D***) and the MFI of CD18, CD40L, and CD69 in the CD4+ gate were analyzed by flow cytometry. ***E***, ***F***, ***G***) The percentages of FOXP3-positive CD4+CD25+ Treg, IL-4-positive CD4 Th2 cells and CD5-positive CD19+CD1d+ Breg cells were analyzed by flow cytometry. Values in the bar graphs are the mean ± SEM. *p < 0.05 and **p < 0.01 compared with vehicle-treated EAE-mice.

Conventional DC (cDC) maturation is a process that involve complex phenotypical changes, including the up-regulation of MHC class II, co-stimulatory and adhesion molecules, the secretion of inflammatory mediators, and altered migratory properties. Plasmocytoid DCs (pDCs) favor the expansion of MOG_35–55_-specific Treg cells and inhibit EAE [21]. Initially, we tested whether *Tn*P interfered during the induction phase with the activation of cDC (CD11c^high^CD11b^high^) or acquisition of suppressive phenotype by pDC (CD11c^int^B220^high^). *Tn*P prophylactic treatment for consecutive 7 days increased the expression of PDL-1 and PDL-2 in pDC compared with EAE vehicle-treated mice (**Fig 6*B***), but did not alter the expression of MHC class II, CD40, CD80 and CD86 in cDC (**Fig 6*A***). The pathogenic function of CD4^+^ T cells was also modulated, *Tn*P targeted specifically the percentage of these cells in spleen (**Fig 6*C***).

The expression of Th cell surface markers of migration and activation, including CD18, CD40L, and CD69 in CD4^+^ splenic T cells were not modulated by *Tn*P (**Fig 6*D***). Then, we explored the possibility that *Tn*P inhibits CD4^+^ T differentiation through an effect on regulatory cells development during EAE differentiation. We found that *Tn*P induced the development of Foxp3^+^ Treg (**Fig 6*E***). Th2 (**Fig 6*F***) cells and CD5^+^CD1d^+^ Breg (**Fig 6*G***) that control the development and activity of encephalitogenic Th1 cells in EAE [22; 23], were not induced by *Tn*P treatment.

**Fig 7:**
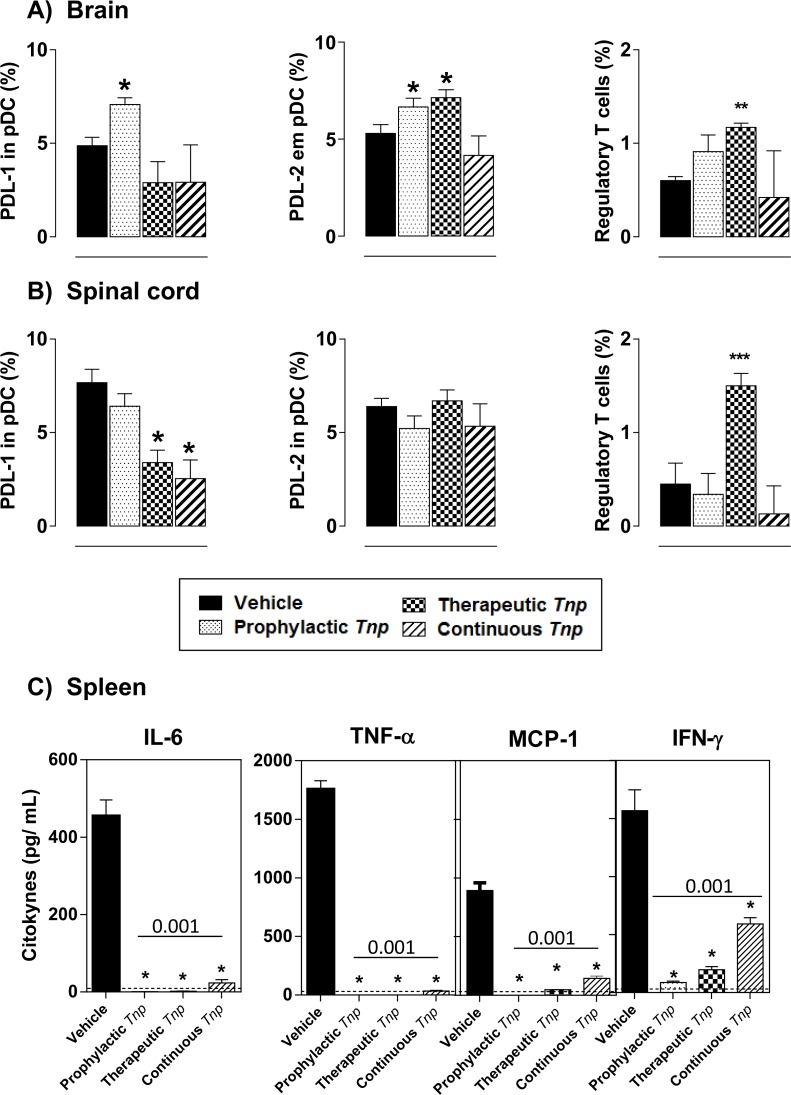
*Tn*P induces the expansion of Treg cells in CNS in an IL-10-dependent manner. At the peak of disease, CNS-infiltrating leukocytes were isolated from pooled brain (***A***) and spinal cord (***B***) homogenates of EAE *WT* or IL-10 *KO* mice treated with vehicle or *Tn*P (n = 5/group), and the percentages of PDL-1- and PDL-2-positive pDC (CD11c+CD45R/B220^low^) or the percentage of FOXP3-positive CD4+CD25+ Treg were evaluated after acquisition of 50,000 events. (***C***) Splenocytes isolated at day 17 were re-stimulated with PMA, ionomycin, and monensin for 4 h, and supernatants were collected to measure IL-6, TNFα, MCP-1, IFN-γ levels by flow cytometry using Cytometric Bead Array. IL-12p70 and IL-10 levels were undetectable. Data represent mean ± SEM. *p < 0.05 and **p < 0.01 and *** *p* < 0.001 compared with vehicle-treated EAE-mice.

Given the importance of immune regulation in the target tissue in EAE, we evaluated the ability of *Tn*P to induce regulatory cells as pDC and Treg in the CNS. Our results in the brain (**Fig 7*A***) during the peak of disease (17) show that prophylactic treatment with *Tn*P increased from 4.9 to 7.0 the percentage of PDL-1-expressing pDC, and prophylactic and therapeutic regimens with *Tn*P increased from 5.3 to 6.7 and 5.3 to 7.1 respectively the percentage of PDL-2-expressing pDC. We also observe that only therapeutic *Tn*P treatment was able to increase the percentage of Treg cells (from 0.6% to 1.2%) and this effect was entirely dependent on IL-10 (**S10 Fig**). In the spinal cord (**Fig 7*B***) in contrast, the therapeutic and continuous *Tn*P treatment induced a decrease of 56% and 65% respectively in the percentage of pDC expressing PDL-1. Also no changes were observed in PDL-2 expression in pDC at day 17 in response to *Tn*P. Regarding to Treg cells, we observe that only the therapeutic regimen promoted high percentage of these cells into the spinal cord in an IL-10 dependent manner (**S10 Fig**).

In order to determine whether cytokines production by effector CD4+ T cells in the peripheral lymphoid organ was modulated in mice treated by *Tn*P, we further investigated the presence of different cytokines in supernatant of splenocytes re-stimulated *in vitro* (**Fig 7*C***). In splenocytes from vehicle-treated EAE mice at day 17, the predominant cytokines induced by PMA re-stimulation were IL-6, TNFα, MCP-1 and IFN-γ. Interestingly, treatment with s.c. *Tn*P at prophylactic, therapeutic and continuous regimens induced significant decrease in the production of all cytokines. Splenocytes of vehicle- or *Tn*P-treated EAE mice produced negligible levels of IL-12p70 (<10.7 pg/ml) or IL-10 (<17.5 pg/ml).

### *Tn*P treatment modulates the encephalitogenic CD4+ Th1 and Th17

**Fig 8:**
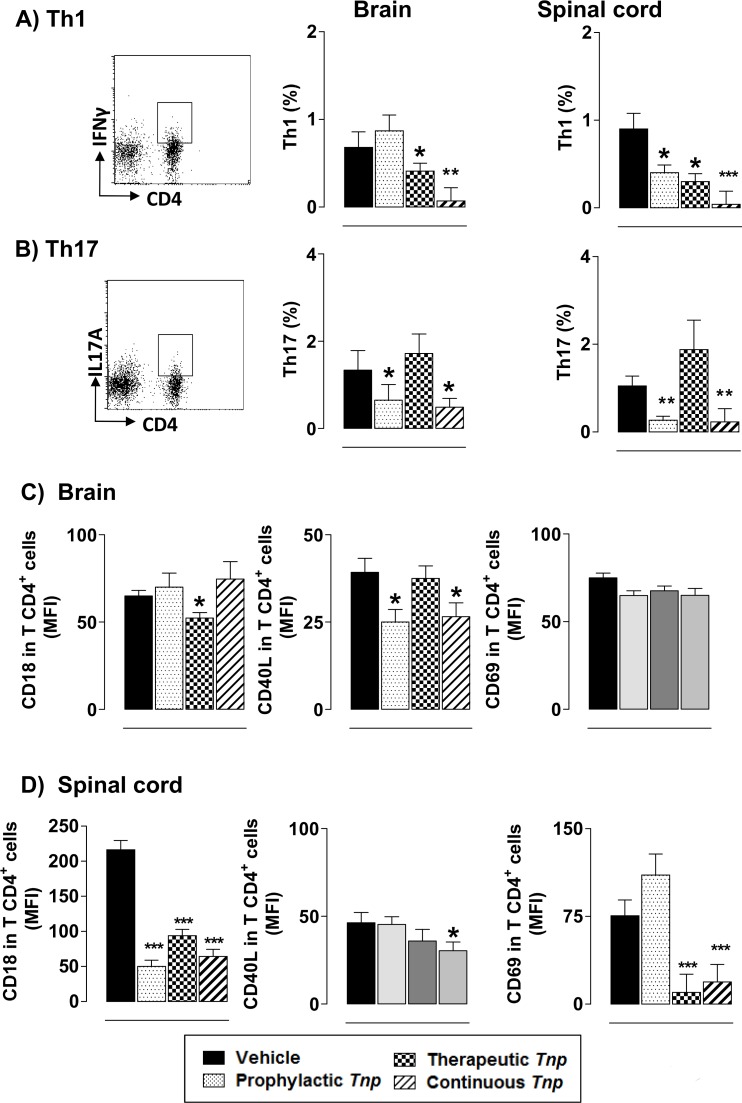
*Tn*P inhibits the expansion of Th1 and Th17 cells in CNS. At the peak of disease, CNS-infiltrating leukocytes were isolated from pooled brain and spinal cord homogenates of EAE mice treated with vehicle or *Tn*P (n = 5/group). Percentages of Th1 (***A***) and Th17 (***B***) cells are shown in the indicated gates after acquisition of 50,000 events. CNS-infiltrating CD4 T lymphocytes were evaluated by the expression (MFI) of CD18, CD40L, and CD69 in brain (***C***) or in spinal cords (***D***). Values in the bar graphs are the mean ± SEM. *p < 0.05 and **p < 0.01 and *** *p* < 0.001 compared with vehicle-treated EAE-mice.

Next, we assessed the ability of *Tn*P to promote the modulation of CD4+ T cell polarization in CNS of EAE treated mice. The effect of *Tn*P in the reduction of IFN-γ-producing Th1 cells (**Fig 8*A***) was observed after the therapeutic (42%) and continuous regimens (80%) in the brain and after the prophylactic (55%), therapeutic (67%) and continuous regimens (80%) in the spinal cord. Only the prophylactic and continuous treatment regimens decreased the percentage of IL-17A-producing Th17 lymphocytes in the brain (45% and 50%, respectively) and in the spinal cord (60% and 60%) (**Fig 8*B***).

In EAE, the up-regulation of ICAM-1 and VCAM-1 on the BBB precedes the perivascular infiltration and the onset of disease, suggesting that their expression is a prerequisite for inflammatory cell entry into the CNS. In the brain (**Fig 8*C***) we observe that the therapeutic *Tn*P treatment induced decrease in the expression of CD18, and the prophylactic and continuous regimens inhibited the expression of CD40L in CD4+ T lymphocytes. The analysis of CD69 expression in CD4+ T cells reveled no differences among groups. In the spinal cord (**Fig 8*D***), inhibition of CD18 expression induced by all regimens of *Tn*P treatment was observed. Only the continuous regimen inhibited the expression of CD40L, and both therapeutic and continuous treatments blocked the expression of CD69. The modulation induced by *Tn*P treatment was not reversed in IL-10 *KO* EAE *Tn*P-treated mice (**S11 Fig**).

### *Tn*P leads to accelerated remyelination in a cuprizone model

**Fig 9:**
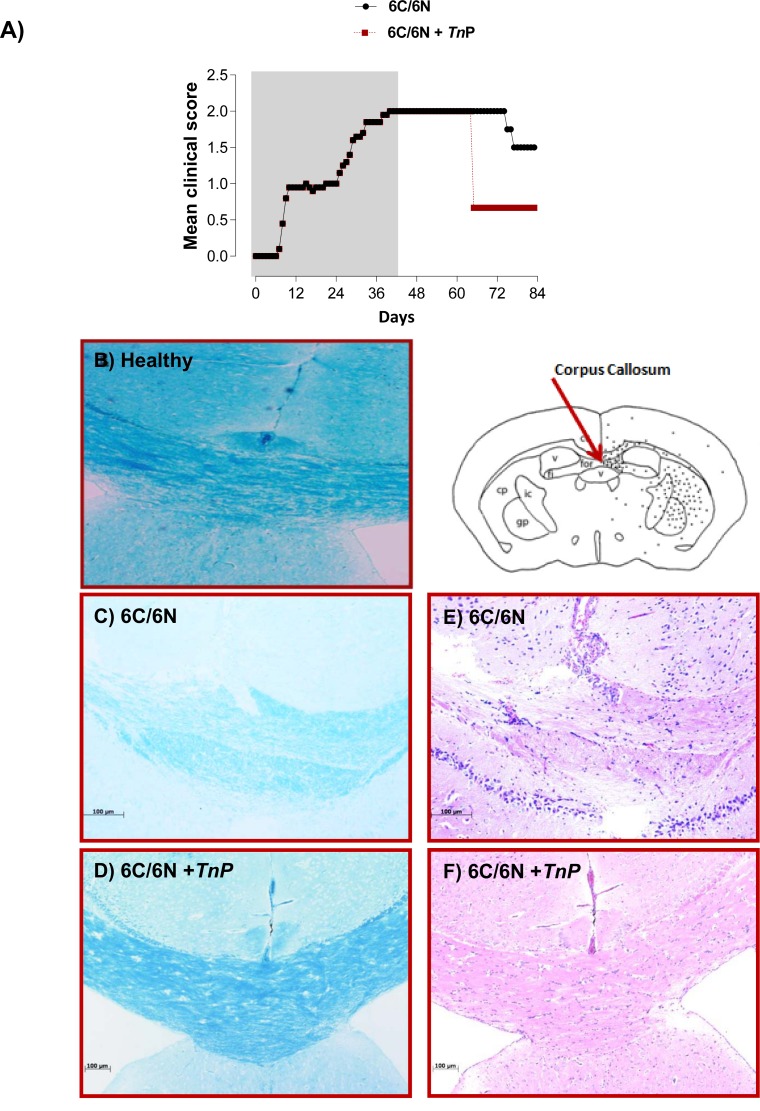
*Tn*P increments the remyelination and inhibits the influx of leukocytes in corpus callosum in cuprizone model. De- and remyelination of the corpus callosum and cortex during cuprizone feeding and *Tn*P treatment. (***A***) C57BL/6 male mice (cuprizone mice were maintained on a normal diet for the duration of 6 weeks under treatment or not with *Tn*P) were scored (0–5) daily for 30 d for evidence of clinical disease signs (n = 15/group). Schematic diagram of brain in coronal section demonstrated normal physiology of mice before cuprizone diet (***B***); an incomplete remyelination of the corpus callosum after 6 weeks of normal diet in cuprizone mice (***C***); and treatment with *Tn*P of cuprizone-mice under normal diet anticipated the strong remyelination at day 66 (***D***). (***B***) Schematic diagram of the mouse brain in coronal section stained with H&E shows accumulation of leukocytes in the corpus callosum and cortex 6 weeks under normal diet in cuprizone-mice (***E***) and absence of infiltration in *Tn*P treated cuprizone mice (***F***).

Next, we evaluated whether *Tn*P has the ability to modulate non-immune cells in the SNC, generating the induction of remyelination, using the toxic model of demyelination induced by cuprizone [24; 25]. First we analyzed the time-course of neurological signs induced by curpizone in Bl6 *WT* cuprizone-mice treated or not with *Tn*P during weeks under normal diet. In **Fig 9*A*** we found that after 6 weeks, cuprizone-mice maintained on a normal diet for further 6 weeks decreased the sign clinical score of 2 to 1.75 at days 76 and 77, reaching a lowest sign clinical score of 1.5 at days 78 to 83. However, the treatment with *Tn*P of cuprizone-mice under normal diet decreased the sign clinical score from 2 to 0.7 at day 66 remaining low until 83. Second, the analysis of the course and extent of demyelination/remyelination in the corpus callosum of brain sections of Bl6 *WT* cuprizone-mice by Luxol fast blue stain revels that mice continuously fed with cuprizone left for another 6 weeks under normal feeding presented spontaneous slight remyelination evident at day 83 (**Fig 9*C***), compared with healthy mice showing normal myelin patterns in the corpus callosum (**Fig 9*B***). The result in **Fig 9*D*** shows that the treatment with *Tn*P of cuprizone-mice under normal diet anticipated the strong remyelination process to day 66.

**Fig 10:**
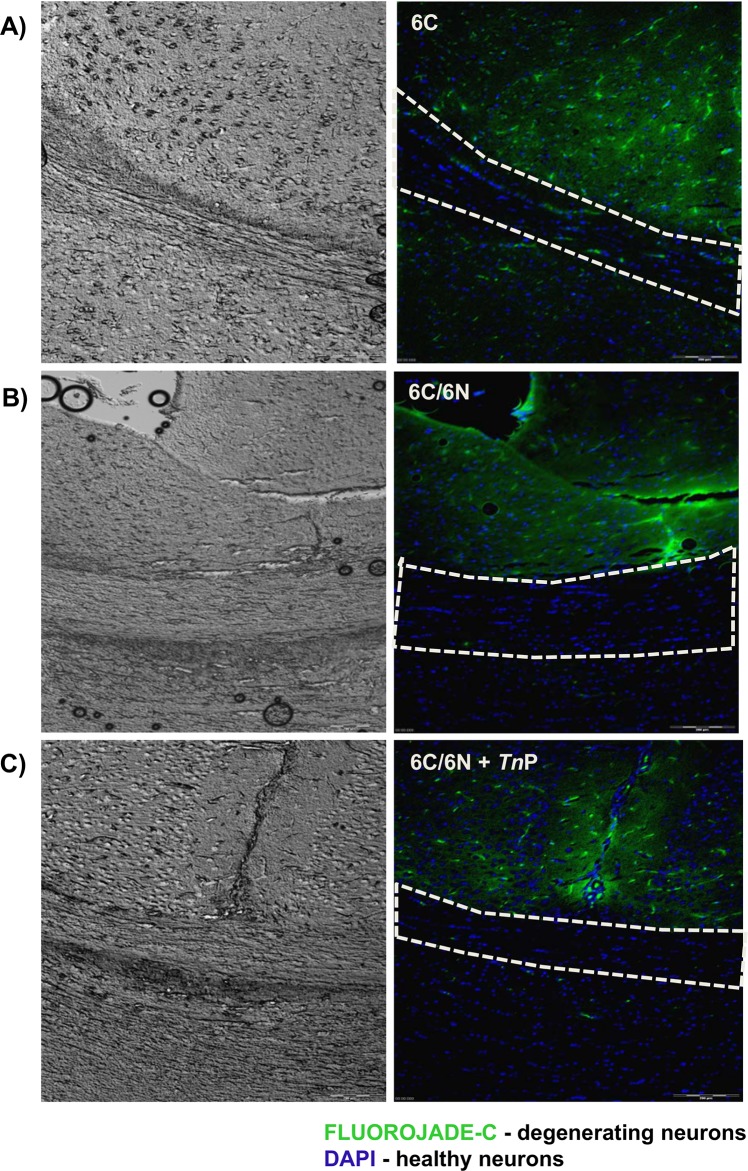
*Tn*P promotes the survival of neurons in corpus callosum. Mice previously fed for 6 weeks on a cuprizone-based diet were maintained for more 6 weeks under standard feed and treated or not with *Tn*P. The immunofluorescent double-staining of brain sections with Fluoro-Jade C (green) and DAPI (blue) revels regenerating neurons after 6 weeks of cuprizone diet (***A***), and healthy neurons in both group of mice under normal diet treated or not (***B***) with *Tn*P (***C***).

Macrophages from the bone marrow are the most numerous cell type accumulating in the cuprizone-mice, coinciding with massive demyelination and initiation of remyelination [26]. In **Fig 9** we confirmed by H&E stain of corpus callosum sections that continuous administration of cuprizone promoted an influx of leukocytes in demyelinating areas (fimbria, fornix, ventricles, and corpus callosum—***E***), however after 6 weeks of normal feeding the recruitment of leukocytes was blocked greatly by *Tn*P treatment (***F***). Next, we investigated whether *Tn*P affected the spontaneous process of remyelination in cuprizone-mice, using the fluorescent marker Fluoro-Jade C (**Fig 10**), which is extremely specific for degenerating neurons [27]. A striking increase in Fluoro-Jade C immunoreactivity was observed in and around the corpus callosum demyelinated areas of cuprizone-mice (***A***), but not after 6 weeks of normal feeding without (***B***) or with *Tn*P treatment (***C***) which in contrast presented an increased number of healthy neurons (in blue).

## Discussion

The results described here indicate that a patented peptide *Tn*P presents as valuable potential first leader candidate to design a new drug to demyelinating conditions as MS, once ameliorated the severity of the clinical signs of MOG-induced EAE, accompanied by inhibition of neuroinflammation and improvement of the remyelination. Our results show that all regimes (prophylactic, therapeutic or continuous) of subcutaneous *Tn*P treatments delayed the onset of maximal symptoms (4 days) and decreased the severity of symptoms by 40% compared to control EAE-mice treated with vehicle alone. Further, prophylactic regimen showed a higher level of disease suppression, and no additive effect was observed in mice submitted to continuous regimen. The lack of additive effect in reducing the symptoms observed in the continuous regimen indicates the benefic effect of the prophylactic *Tn*P regimen and implies that the action of the *Tn*P administered at the time of disease induction is crucial for both the suppression of trafficking of encephalitogenic T cells across BBB at the effector phase and for the suppression of *in situ* reactivation of effector CD4 T cells in the spinal cord. This view is consistent with our observation that mice treated with *Tn*P have dramatically lower numbers of CNS infiltrating cells than control EAE-mice.

We demonstrated that the therapeutic effects of the *Tn*P in delaying the onset of severe symptoms or decreasing the mean symptoms intensity were dependent on IL-10 as described by other [28–30]. Also the control of neuroinflammation induced by the prophylactic and therapeutic regimens may be associated with the induction of peripheral and located in the CNS of regulatory cells as pDC and CD4+CD25+Foxp3+ Treg cells. These results highlight the impact of *Tn*P treatment on regulatory cell response and on the phenotype or composition of the splenic CD11c^+^ DC compartment. The induction of inhibitory molecules in pDC at the induction phase of EAE emphasizes that *Tn*P modulates the transition of DC from immunogenic to tolerogenic state of activation. The immune modulation characterized by the suppression of antigen presentation and co-stimulatory molecules has been observed in EAE mice treated with statins, simvastatin and atorvastatin compounds [31–33]. Glatiramer acetate modifies the immune response by inducing a shift in T cell populations from Th1 to Th2 cells, increasing regulatory T cells, inhibiting the activation of myelin basic protein (MBP)-specific T cells, directly inhibiting antigen presenting cells and reducing IFN-γ levels [34].

Overall, our results show that *Tn*P via IL-10 increases the population of regulatory cells in the brain and spinal cord of EAE-treated mice, and even with the ongoing disease the therapeutic treatment with *Tn*P is able to generate Treg cells in the CNS. The increased number of Tregs in the CNS induced by therapeutic *Tn*P regimen may be responsible for reduced CNS leukocyte inflammation through mechanisms such as reduced production of pro-inflammatory cytokines and chemokines in spleen (IL-6, TNFα, IFN-γ, and MCP-1). Our results described here are consistent with previous reports showing the important role of IL-10 in both the induction of regulatory cells and their effector function [35]. Treg cell is one of mechanisms involved in the suppressive effect of glatiramer acetate of MS [36].

However, the continuous regimen of treatment with *Tn*P inhibited the neuroinflammation independent on IL-10, showing that the application of the *Tn*P during induction and effector phases creates a protective effect without acting as an overt immunosuppressant (splenocytes of vehicle- or *Tn*P-treated EAE mice produced negligible levels of IL-10 after re-stimulation). One advantage to *Tn*P, once immunosuppression along with cardiotoxicity is one of the side effects of biological medicinal products in use for MS [37]. Immunosuppression generates in some patients an increased susceptibility to the development of opportunistic viral infections such as progressive multifocal leukoencephalopathy (PML). PML is a severe and well-documented complication of natalizumab treatment [38; 39].

The importance for MS of pro-inflammatory signaling cascades, as well as leukocyte–endothelium interactions, has been demonstrated in the MOG-induced EAE [40]. Also, strategies to block either interactions between leukocytes and vascular endothelial cells and CNS or systemic inflammation reduce the pathogenesis of MS. Natalizumab is a humanized monoclonal antibody to α4 integrin (VLA-4 is a heterodimeric integrin composed of α4 and β1 subunits) on lymphocytes and some myeloid cells as monocytes which binds to the vascular cell adhesion molecule (VCAM)-1 receptor on endothelial cells thereby inhibiting transmigration of these cells through capillary endothelium into CNS [41]. Analysis of inflammatory cell subpopulations showed that MMP-9-producing macrophages and microglia were significantly lower in spinal cords of EAE-mice treated with all *Tn*P regimens than in untreated mice. We have demonstrated that *Tn*P inhibited the movement of macrophages into the CNS at the peak of disease and was able to minimize the population of activated microglial cells by mechanisms independent on IL-10, suggesting that in addition to its ability to suppress the production of specific chemokine for monocytes, *Tn*P can also act directly on these cells minimizing their activation response. Inhibitors of trypsin are of widespread occurrence in different taxa and are representative of many established structural classes, including Kunitz, Kazal and Bowman-Birk. We can speculate that in addition to serine protease inhibition capacity of *Tn*P, protease inhibitors also often possess other intrinsic properties that contribute to termination of the inflammatory process, including modulation of cytokine expression, signal transduction and tissue remodeling [42].

*Tn*P treatment not only suppresses the expression of CD18 (β2 integrin), minimizing the arrival of CD4+ T cells in the CNS at the peak of disease, but also decreases the expression of co-stimulatory and activating molecules (CD40L or CD69, respectively), making the T lymphocyte less auto-reactive in the CNS as has been described [43]. Interestingly, our data included a interesting result, showing that all regimens of *Tn*P treatment were able to minimize the population of pathogenic Th1 lymphocytes in the CNS, however the Th17 cells were only decreased by prophylactic (and continuous) treatment. Further Treg cells in CNS were only induced by therapeutic treatment. One hypothesis that could explain the difference in the ability of *Tn*P to inhibit Th1 rather than Th17 cells in CNS is the preferential repertoire of chemokine receptors and integrins that guide the entry of Th1 and Th17 cells into the CNS [44]. A second hypothesis is that IL-17-producing Foxp3-positive regulatory T cells may arise from naive Treg cells in the presence of inflammatory cytokines and still retain a suppressive function [45; 46]. Finally, our data corroborate the finding showing that IFN-β treatment is ineffective to a subset of RRMS patients with Th17-skewed disease. This phenomenon was supported by observations of Axtell et al. [47], which identified that mice with Th1-induced EAE benefit from IFN-β treatment with a reduction in the degree of disability, whereas mice with Th17-induced EAE do not respond, and their disease worsens. Our results emphasize the high degree of complexity in determining the biomarkers of MS phenotypes.

One of the challenges in MS research is to understand the shortcomings of the remyelination process and develop strategies to restore myelination. Neuroprotection through remyelination become a key therapeutic aim in MS. The wealth of other new drugs designed to reduce MS relapses, which are in clinical trial, awaiting licensing, or that have received licensing in some countries, have not been shown to affect disease progression or induce remyelination. The results of phase 2 trial in both relapsing remitting and/or secondary progressive MS with the neutralizing monoclonal antibody Opicinumab against the protein LINGO present in nerve cells and oligodendrocytes showed that the neuroreparative anti-LINGO-1 missed its primary end point. The treatment failed to improve disability, physical or cognitive function [ClinicalTrials.gov Identifier: NCT01721161].

A striking supportive evidence that shows that *Tn*P can restores normal axonal health and prevents neurodegeneration came from the study of 8 week old C57BL/6 mice fed with a cuprizone-supplemented diet for 6 weeks. This model is particularly useful for studying demyelination and remyelination, and their relation to axonal loss. Our results show that *Tn*P accelerates the remyelination process through the inhibition of the leukocytes infiltration. Recently, Liu et al. confirmed the essential role of neutrophil inflammation in demyelination process, along with nitrative stress associated with nNOS activity in CNS-resident cells. They found that CXCR2-positive Gr1-positive myeloid cells are required for new cycles of oligodendrocyte cell loss and demyelination after cuprizone challenge [48].

The emergence of immunomodulatory drugs as small molecules allows oral administration, which circumvent the difficulties associated with intravenous biological products, including the generation of neutralizing antibodies and low medication adherence. Recently, Thell et al [49] confirmed the benefic effect of the prophylactic and therapeutic oral administration of plant-derived peptide cyclotide [T20K]kB1 in EAE-mice, reducing the polarization of pathogenic Th17 cells and the rate of relapse which potently ameliorated the EAE symptoms. Although our *in vitro* data confirm that *Tn*P is functionally and structurally resistant to the extreme conditions as acidic pH and severe heat treatment, we can assume the use of pharmacological and biotechnological alternatives or design of delivery directed-systems, which might protect *Tn*P peptide against possible enzymatic degradation.

In conclusion, our results indicate that application of *Tn*P during or between acute attacks or even continuously generates systemic and CNS localized effects that result in inhibition of traffic of inflammatory leukocyte to CNS and demyelination, and lead to improvement of remyelination. These findings support the beneficial effects of *Tn*P and provides a new therapeutic opportunity for the treatment of MS.

## Supporting information

S1 Fig Amino acid sequence and three-dimensional structure of *Tn*P. The analysis of amino acid sequence of *Tn*P (P13821401, C_63_H_114_N_22_O_13_S_4_, and purity of 97,3%) was done by a MALDI-ToF/PRO instrument (G&E Healthcare—Sweden). The three-dimensional structure was constructed by homology modeling using as templates homologous proteins uncovered by Protein Data Bank screening, based on the structure of antitrypsin (PDB code: 1ATU).
(TIF)

S2 Fig EAE model and *Tn*P treatment. C57BL/6 *WT* or IL-10 *KO* mice (n = 15/group) immunized with (MOG)_35-55_ peptide in incomplete Freund’s adjuvant added with *M*. *tuberculosis* were injected 2 times with *Pertussis* toxin after immunization. Mice was scored (0–5) daily for 30 d for evidence of clinical disease (n = 15/group). Mice was treated with 3 mg/kg of *Tn*P diluted in 0.9% saline every other day starting at the day of immunization as following: day 0 to 9 (Prophylactic), from day 10 to 19 (Therapeutic) or from day 0 to 19 (Continuous). The EAE controls were injected with 0.9% saline alone (Vehicle). Mice were killed at days 7, 17 and 30 for analysis.
(TIF)

S3 Fig Cuprizone-model of demyelination and *Tn*P treatment. Demyelination was induced by feeding 8–10 week old male C57BL/6 mice with a diet containing 0.2% (wt/wt) cuprizone mixed into a ground Breeder Chow 2000 for up to 6 consecutive weeks. Mice was daily monitored for clinical signs and killed at 6 weeks of diet to determine neuropathology and to conduct histological analyzes. After 6 weeks, healthy control or cuprizone mice were maintained on a normal diet for further 6 weeks. For the therapeutic study, groups of at least five mice were s.c. injected with 100 μl of *Tn*P at dose of 3 mg/Kg for 3 alternate days per week and killed after 1, 2, 3, 4, 5 or 6 weeks of normal feeding. Clinical sign scores of neurological disorder were daily assigned as follows: 1, tail limpness; 2, impaired righting reflex; 3, hind limb paralysis; 4, hind- and forelimb paralysis; 5, death.
(TIF)

S4 Fig Dose-response curve of *Tn*P treatment. C57BL/6 *WT* EAE-mice was treated with different doses of *Tn*P (0.2, 0.4, 0.8, 1.5, and 3 mg/kg) diluted in 0.9% saline every other day starting at the day of immunization during days 0 to 9 (Prophylactic). The EAE controls were injected with 0.9% saline alone (vehicle). Mice was scored (0–5) daily for 30 days for evidence of clinical disease signs, and the day of onset (***A***) and the maximal score (***B***) were determined. Paraffin-embedded sections of spinal cord were removed on day 17 and stained with Luxol fast blue for quantification of demyelination (***C***). Data represent mean ± SEM. * *p* < 0.05, ** *p* < 0.01, and *** *p* < 0.001 compared with *WT* vehicle-treated EAE-mice.
(TIF)

S5 Fig *Tn*P treatment controls MMP-9 activity, the recruitment of cells to SNC and improves the body weight. Gelatin gel zymography of pooled tissue homogenates of spinal cords from *Tn*P treated EAE-mice shows decrease of active and pro-form of MMP-9 after treatments (***A***). Paraffin-embedded sections of spinal cord were removed on day 17 and stained with hematoxylin and eosin (H&E) for quantification of inflammation (***B***). Mice was weighed daily and the percentage of increment of weight compared to normal weight of healthy mice was evaluated (***C***). Data represent mean ± SEM. * *p* < 0.05; ** *p* < 0.01. *** *p* < 0.001 compared with *WT* vehicle-treated EAE-mice.
(TIF)

S6 Fig The inhibition of leukocyte infiltration induced by *Tn*P is independent on IL-10. Quantification of brain (***A***) and spinal cord (***B***) leukocyte infiltrate in pooled tissue homogenates collected at days 17 and 30 from *WT* and IL-10 *KO Tn*P-treated EAE mice or vehicle treated mice (n = 5/group). Data represent mean ± SEM. *p < 0.05 compared with vehicle-treated EAE-mice.
(TIF)

S7 Fig *Tn*P inhibits the infiltration of leukocytes to CNS and demyelination independent on IL-10. Quantification of spinal cord cellular infiltrates and demyelination (***B***) in *WT* (***A***) or IL-10 *KO* (***B***) vehicle- or therapeutically *Tn*P-treated EAE mice (n = 5/group). Spinal cords from healthy and EAE mice treated with vehicle or *Tn*P were removed on the peak of disease (17) and stained in with H&E in the upper panels or Luxol fast blue in the lower ones. The quantification of cells and demyelination were evaluated blindly. Representative sections are shown. Data represent mean ± SEM. *p < 0.05 compared with vehicle-treated EAE-mice.
(TIF)

S8 Fig The reduction of microglia expansion and infiltration of macrophages is independent on IL-10. At days 17 and 30 post immunization, CNS-infiltrating leukocytes were isolated from pooled brain (***A***) and spinal cord (***B***) homogenates of *WT* or IL-10 *KO* EAE mice treated with vehicle or *Tn*P (n = 5/group), and the percentages of microglia (CD11b^low^CD45^low^) and infiltrating macrophages (CD11b^high^CD45^high^) as depicted in dot plot were analyzed by flow cytometry after acquisition of 50,000 events. Data represent mean ± SEM. *p < 0.05 compared with vehicle-treated EAE-mice.
(TIF)

S9 Fig The inhibition of the gelatinase activity induced by *Tn*P is independent on IL-10. Gelatin gel zymography of pooled tissue homogenates of spinal cords at day 17 from *WT* or IL-10 *KO* EAE mice treated with vehicle or *Tn*P and healthy mice (n = 5/group) shows active and pro-form of MMP-9 (***A***). Bar graphs (***B*** and ***C***) show densitometry quantification of representative gel zymography data. rMMP-9 was used as standard. Data represent mean ± SEM. *p < 0.05 compared with vehicle-treated EAE-mice.
(TIF)

S10 Fig *Tn*P induces T regulatory cells dependent on IL-10. At the peak of disease, CNS-infiltrating leukocytes were isolated from pooled brain (***A***) and spinal cord (***B***) homogenates of *WT* or IL-10 *KO* EAE mice treated with vehicle or *Tn*P (n = 5/group), and the percentages of PDL-1- and PDL-2-positive pDC (CD11c+CD45R/B220^low^) or the percentage of FOXP3-positive CD4+CD25+ Treg were evaluated after acquisition of 50,000 events. Data represent mean ± SEM. *p < 0.05 compared with vehicle-treated EAE-mice and # *p* < 0.05 compared with *WT Tn*P-treated EAE-mice.
(TIF)

S11 Fig *Tn*P inhibits the expansion of Th1 and Th17 cells in CNS independent on IL-10. At day 17, CNS-infiltrating leukocytes were isolated from pooled brain and spinal cord homogenates of *WT* or IL-10 *KO* EAE mice treated with vehicle or *Tn*P (n = 5/group). Percentages of Th1 (***A***) and Th17 (***B***) cells are shown in the indicated gates after acquisition of 50,000 events. CNS-infiltrating CD4 T lymphocytes were evaluated by the expression (MFI) of CD18, CD40L, and CD69 in brain (***C***) or in spinal cords (***D***). Values in the bar graphs are the mean ± SEM. *p < 0.05 compared with vehicle-treated EAE-mice.
(TIF)
