# Supplementary figures and images for: Multiple functional therapeutic effects of TnP: A small stable synthetic peptide derived from fish venom in a mouse model of multiple sclerosis
Source: PLoS One. 2017 Feb 24;12(2):e0171796. doi: 10.1371/journal.pone.0171796 (PMC5325231; doi:10.1371/journal.pone.0171796)

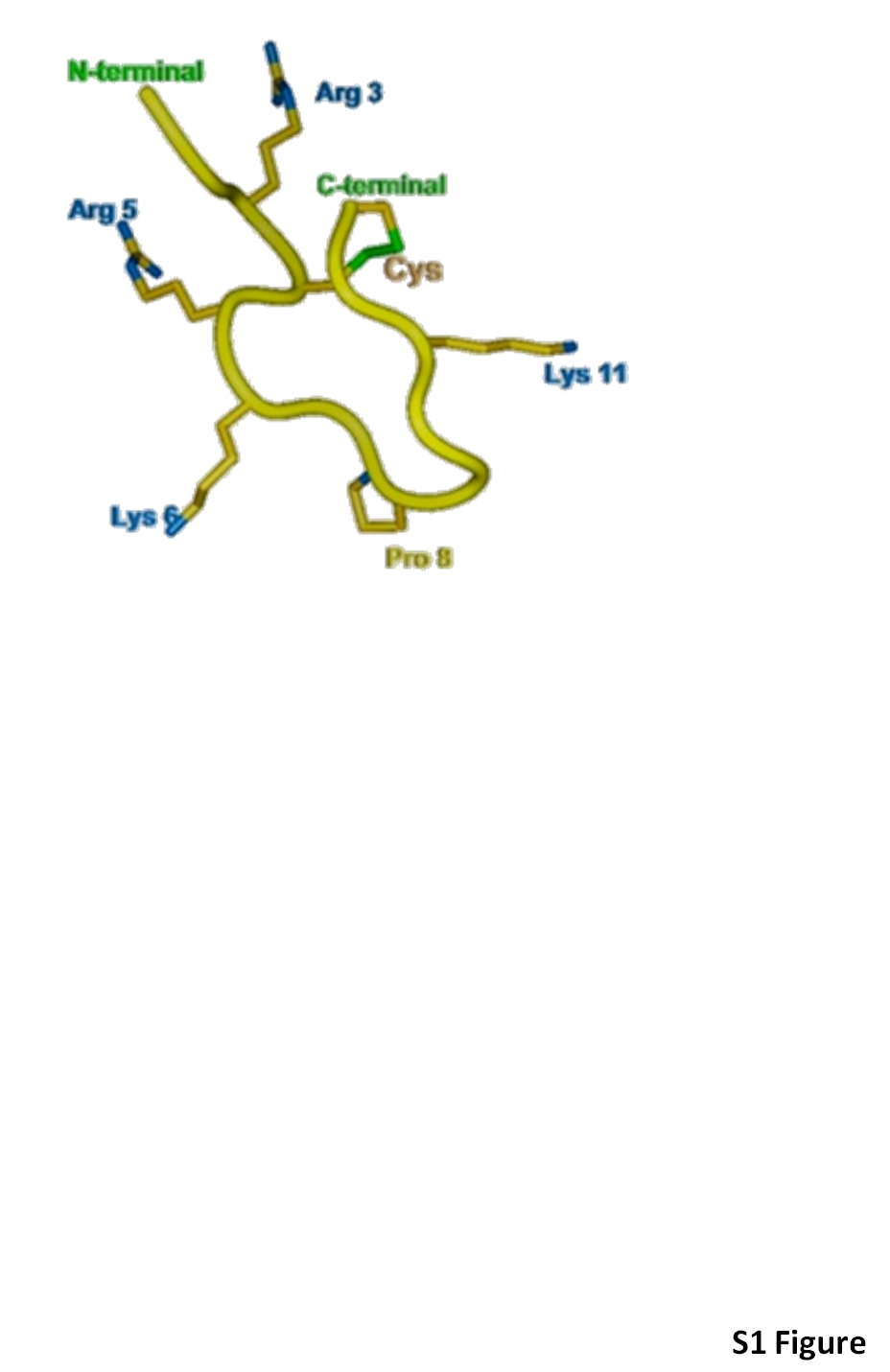

Supplement: S1 Fig — The analysis of amino acid sequence of TnP (P13821401, C63H114N22O13S4, and purity of 97,3%) was done by a MALDI-ToF/PRO instrument (G&E Healthcare—Sweden). The three-dimensional structure was constructed by homology modeling using as templates homologous proteins uncovered by Protein Data Bank screening, based on the structure of antitrypsin (PDB code: 1ATU). (TIF) [file pone.0171796.s001.tif]

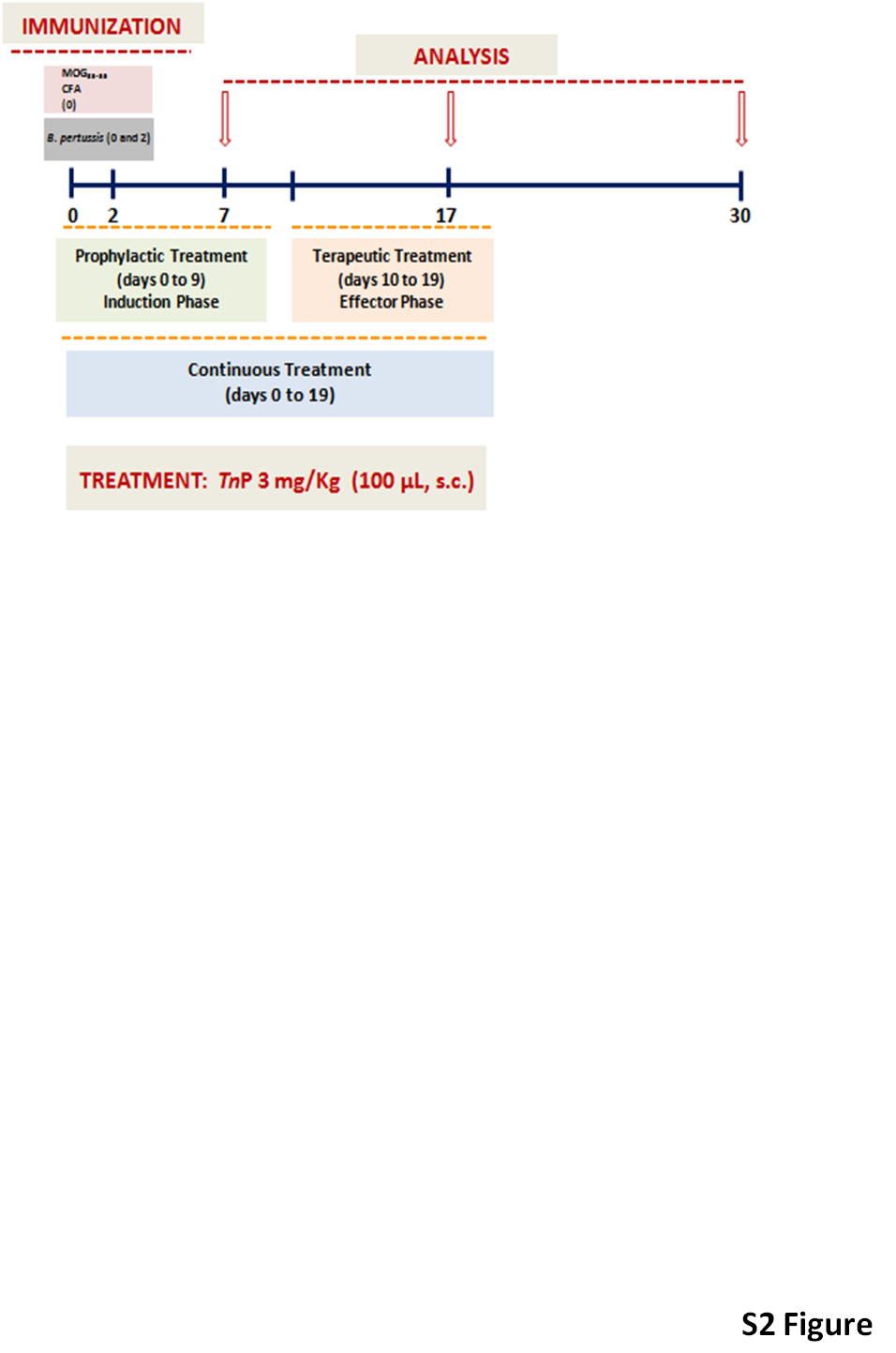

Supplement: S2 Fig — C57BL/6 WT or IL-10 KO mice (n = 15/group) immunized with (MOG)35-55 peptide in incomplete Freund’s adjuvant added with M. tuberculosis were injected 2 times with Pertussis toxin after immunization. Mice was scored (0–5) daily for 30 d for evidence of clinical disease (n = 15/group). Mice was treated with 3 mg/kg of TnP diluted in 0.9% saline every other day starting at the day of immunization as following: day 0 to 9 (Prophylactic), from day 10 to 19 (Therapeutic) or from day 0 to 19 (Continuous). The EAE controls were injected with 0.9% saline alone (Vehicle). Mice were killed at days 7, 17 and 30 for analysis. (TIF) [file pone.0171796.s002.tif]

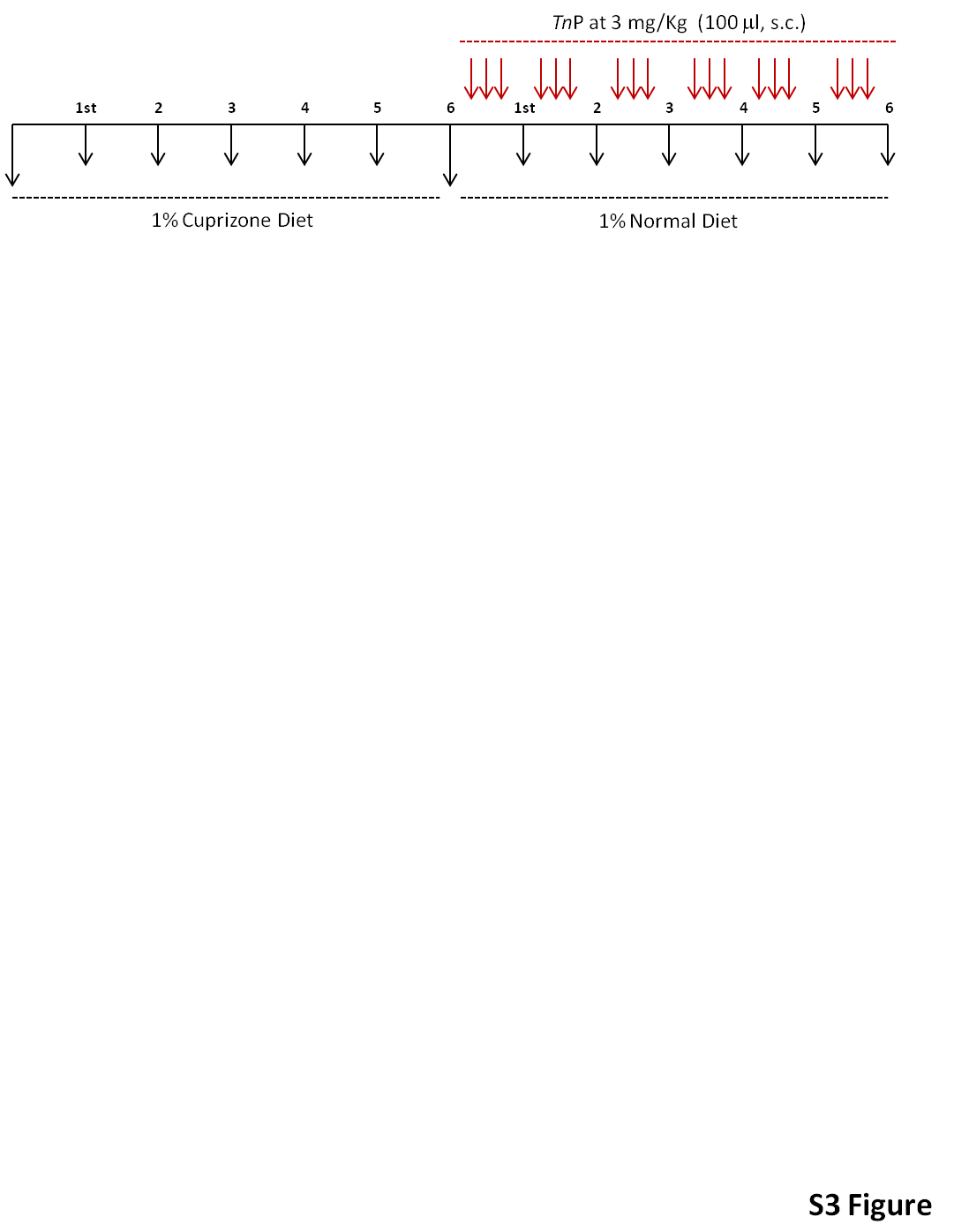

Supplement: S3 Fig — Demyelination was induced by feeding 8–10 week old male C57BL/6 mice with a diet containing 0.2% (wt/wt) cuprizone mixed into a ground Breeder Chow 2000 for up to 6 consecutive weeks. Mice was daily monitored for clinical signs and killed at 6 weeks of diet to determine neuropathology and to conduct histological analyzes. After 6 weeks, healthy control or cuprizone mice were maintained on a normal diet for further 6 weeks. For the therapeutic study, groups of at least five mice were s.c. injected with 100 μl of TnP at dose of 3 mg/Kg for 3 alternate days per week and killed after 1, 2, 3, 4, 5 or 6 weeks of normal feeding. Clinical sign scores of neurological disorder were daily assigned as follows: 1, tail limpness; 2, impaired righting reflex; 3, hind limb paralysis; 4, hind- and forelimb paralysis; 5, death. (TIF) [file pone.0171796.s003.tif]

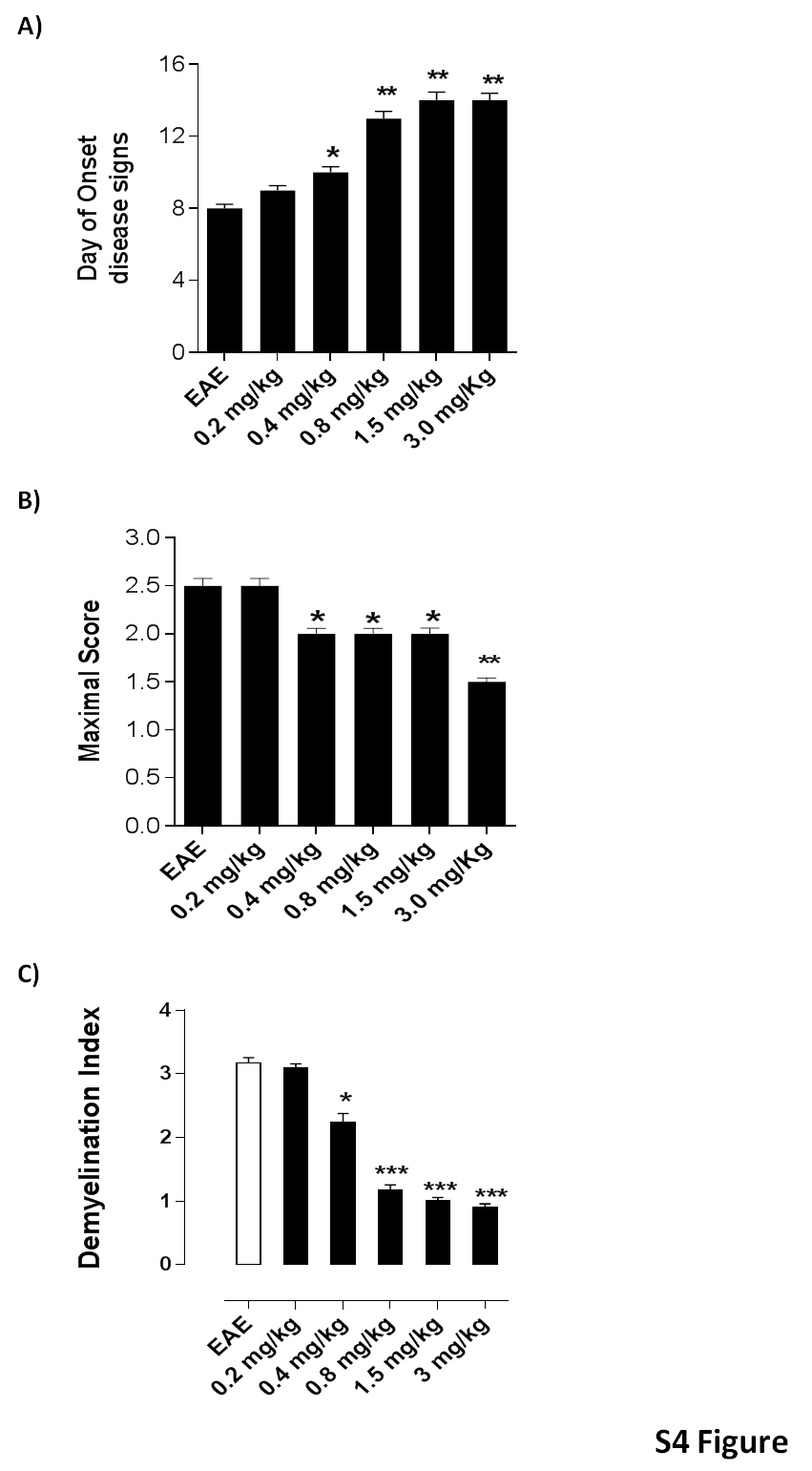

Supplement: S4 Fig — C57BL/6 WT EAE-mice was treated with different doses of TnP (0.2, 0.4, 0.8, 1.5, and 3 mg/kg) diluted in 0.9% saline every other day starting at the day of immunization during days 0 to 9 (Prophylactic). The EAE controls were injected with 0.9% saline alone (vehicle). Mice was scored (0–5) daily for 30 days for evidence of clinical disease signs, and the day of onset (A) and the maximal score (B) were determined. Paraffin-embedded sections of spinal cord were removed on day 17 and stained with Luxol fast blue for quantification of demyelination (C). Data represent mean ± SEM. * p < 0.05, ** p < 0.01, and *** p < 0.001 compared with WT vehicle-treated EAE-mice. (TIF) [file pone.0171796.s004.tif]

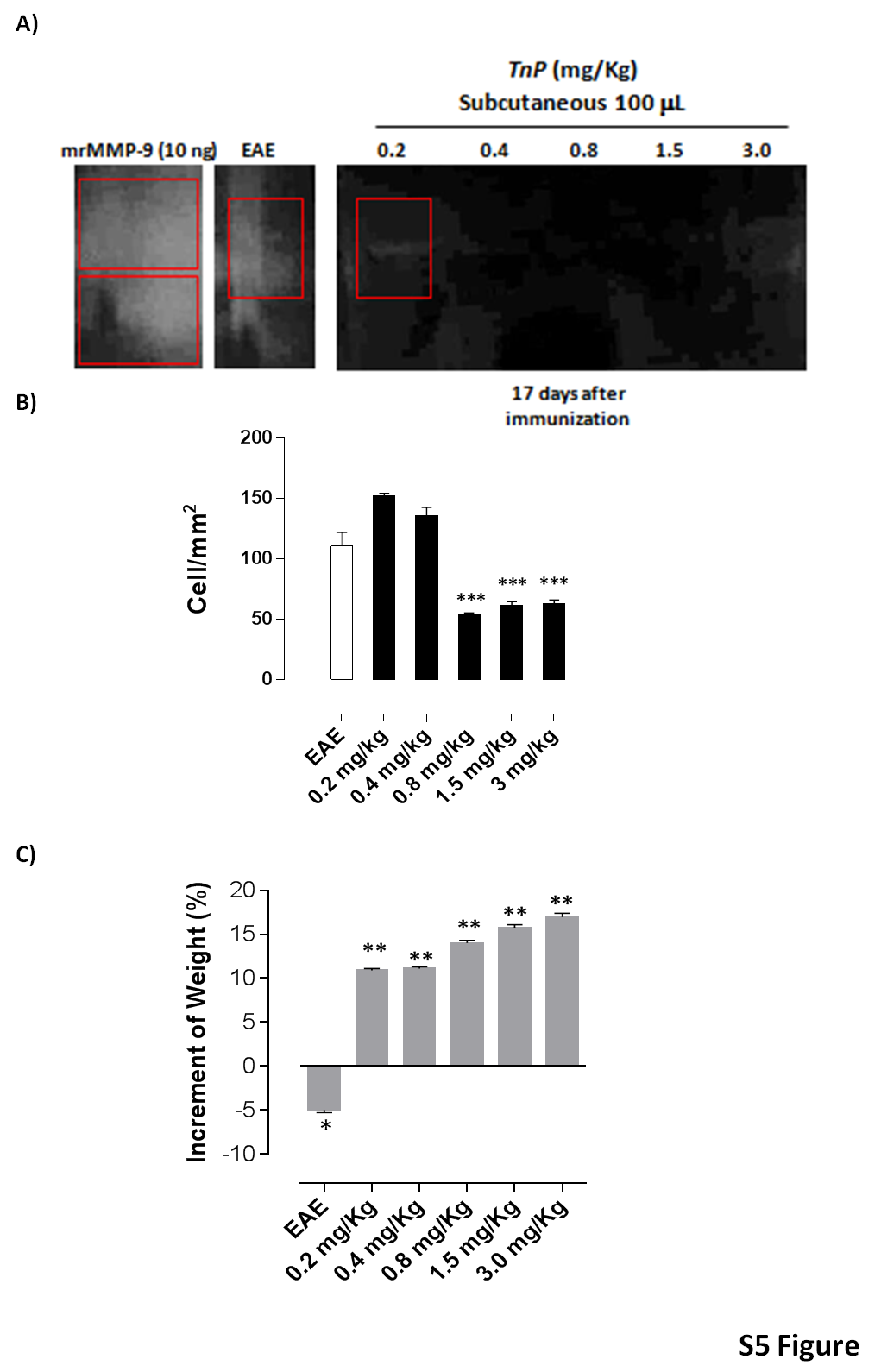

Supplement: S5 Fig — Gelatin gel zymography of pooled tissue homogenates of spinal cords from TnP treated EAE-mice shows decrease of active and pro-form of MMP-9 after treatments (A). Paraffin-embedded sections of spinal cord were removed on day 17 and stained with hematoxylin and eosin (H&E) for quantification of inflammation (B). Mice was weighed daily and the percentage of increment of weight compared to normal weight of healthy mice was evaluated (C). Data represent mean ± SEM. * p < 0.05; ** p < 0.01. *** p < 0.001 compared with WT vehicle-treated EAE-mice. (TIF) [file pone.0171796.s005.tif]

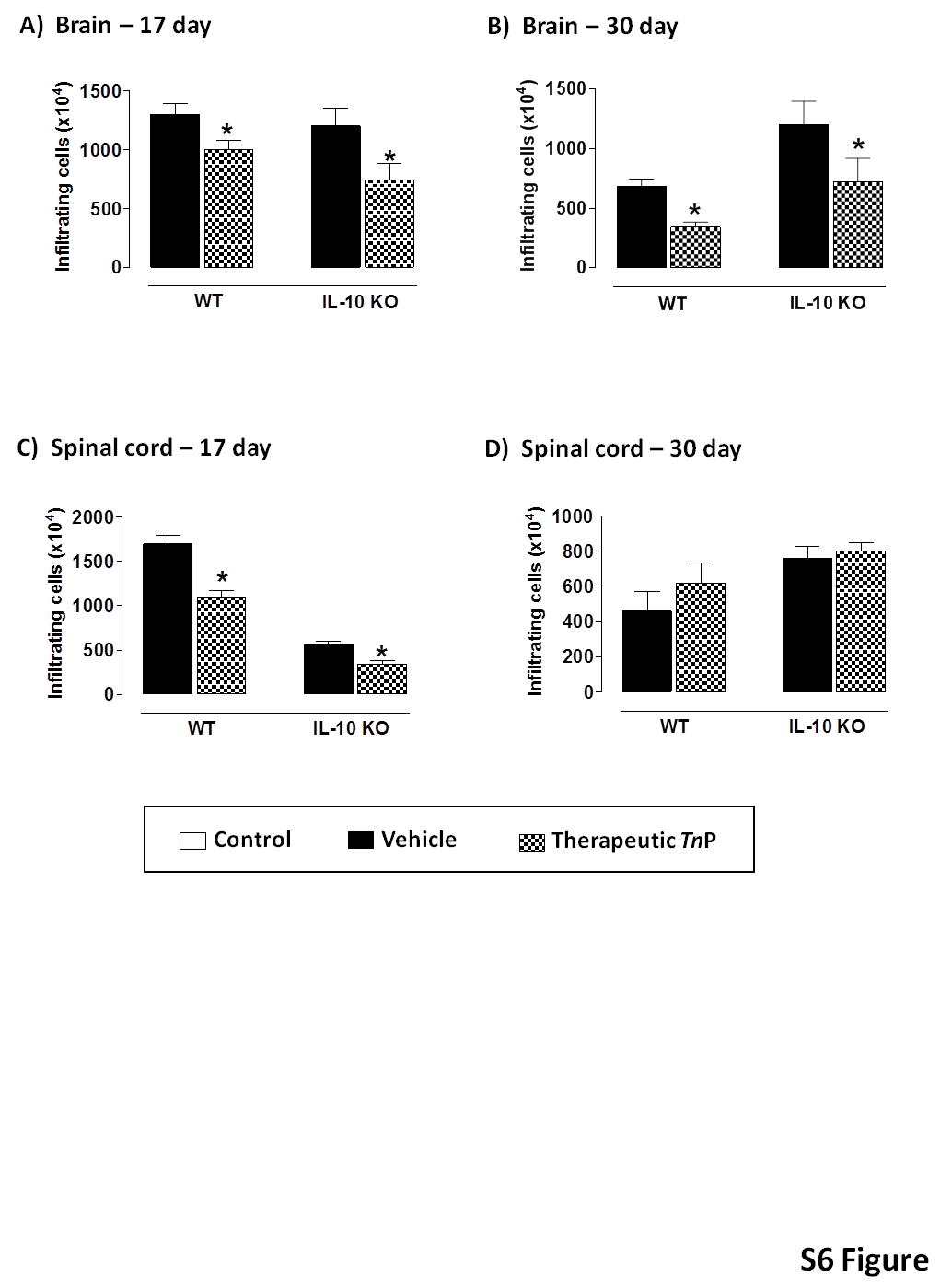

Supplement: S6 Fig — Quantification of brain (A) and spinal cord (B) leukocyte infiltrate in pooled tissue homogenates collected at days 17 and 30 from WT and IL-10 KO TnP-treated EAE mice or vehicle treated mice (n = 5/group). Data represent mean ± SEM. *p < 0.05 compared with vehicle-treated EAE-mice. (TIF) [file pone.0171796.s006.tif]

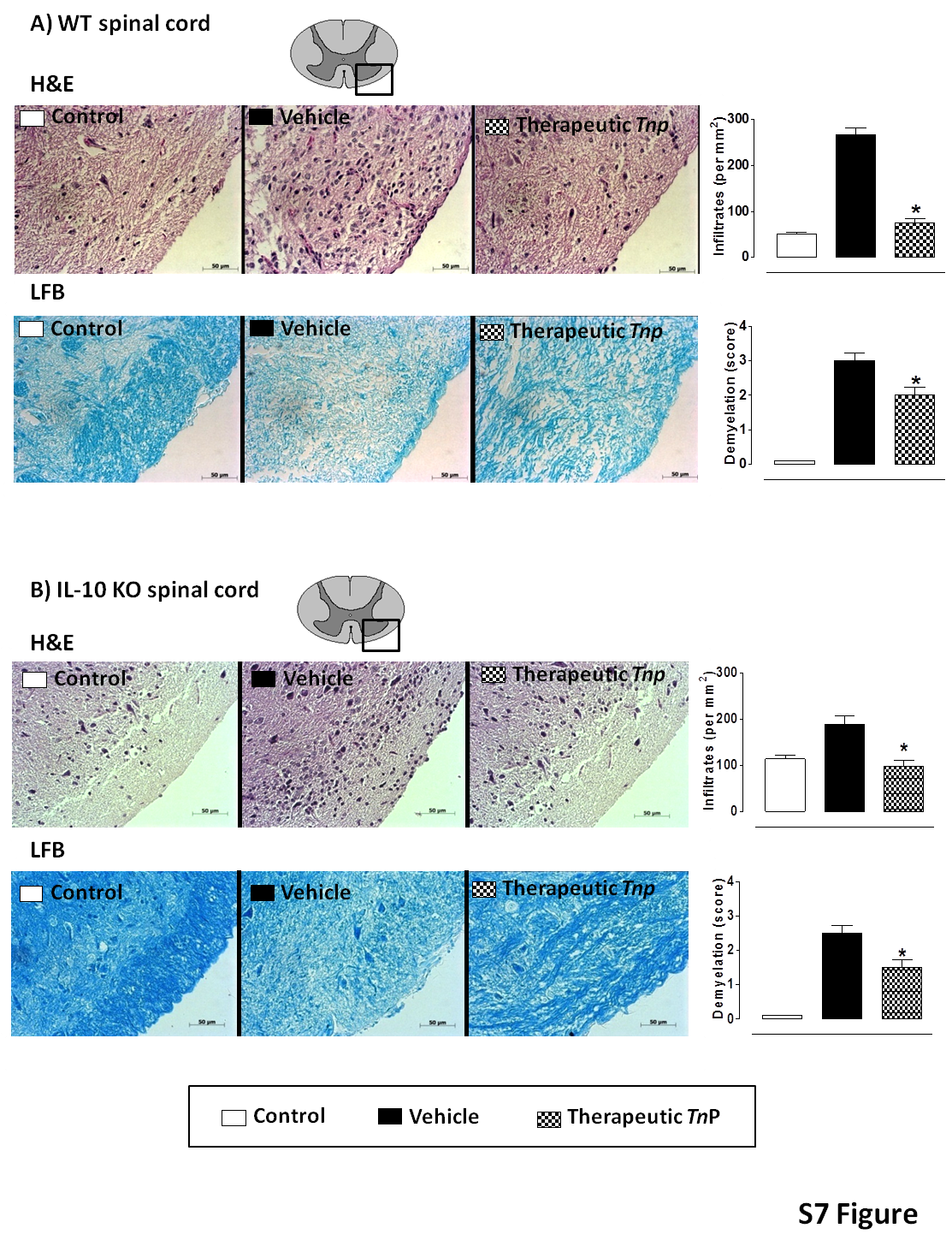

Supplement: S7 Fig — Quantification of spinal cord cellular infiltrates and demyelination (B) in WT (A) or IL-10 KO (B) vehicle- or therapeutically TnP-treated EAE mice (n = 5/group). Spinal cords from healthy and EAE mice treated with vehicle or TnP were removed on the peak of disease (17) and stained in with H&E in the upper panels or Luxol fast blue in the lower ones. The quantification of cells and demyelination were evaluated blindly. Representative sections are shown. Data represent mean ± SEM. *p < 0.05 compared with vehicle-treated EAE-mice. (TIF) [file pone.0171796.s007.tif]

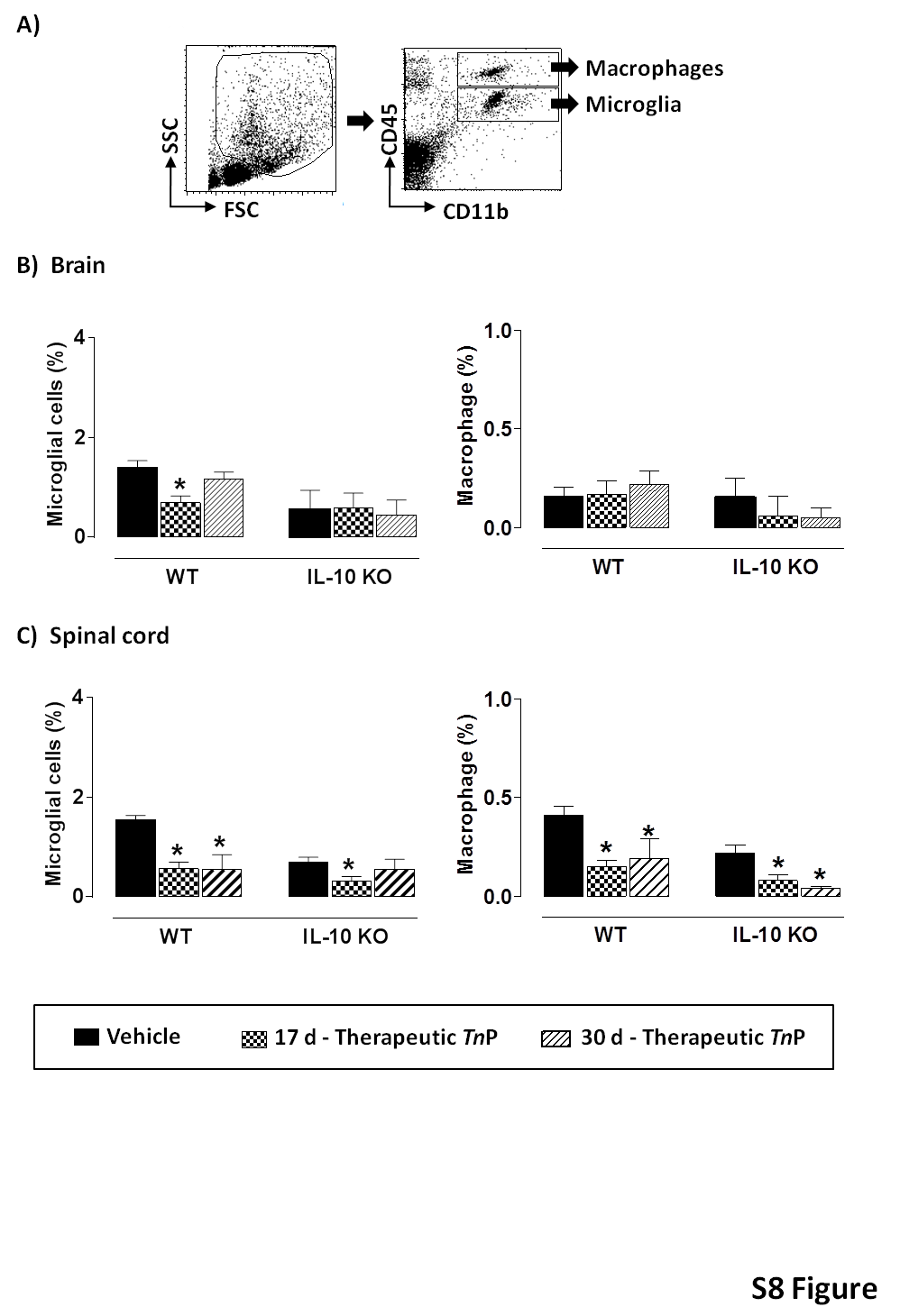

Supplement: S8 Fig — At days 17 and 30 post immunization, CNS-infiltrating leukocytes were isolated from pooled brain (A) and spinal cord (B) homogenates of WT or IL-10 KO EAE mice treated with vehicle or TnP (n = 5/group), and the percentages of microglia (CD11blowCD45low) and infiltrating macrophages (CD11bhighCD45high) as depicted in dot plot were analyzed by flow cytometry after acquisition of 50,000 events. Data represent mean ± SEM. *p < 0.05 compared with vehicle-treated EAE-mice. (TIF) [file pone.0171796.s008.tif]

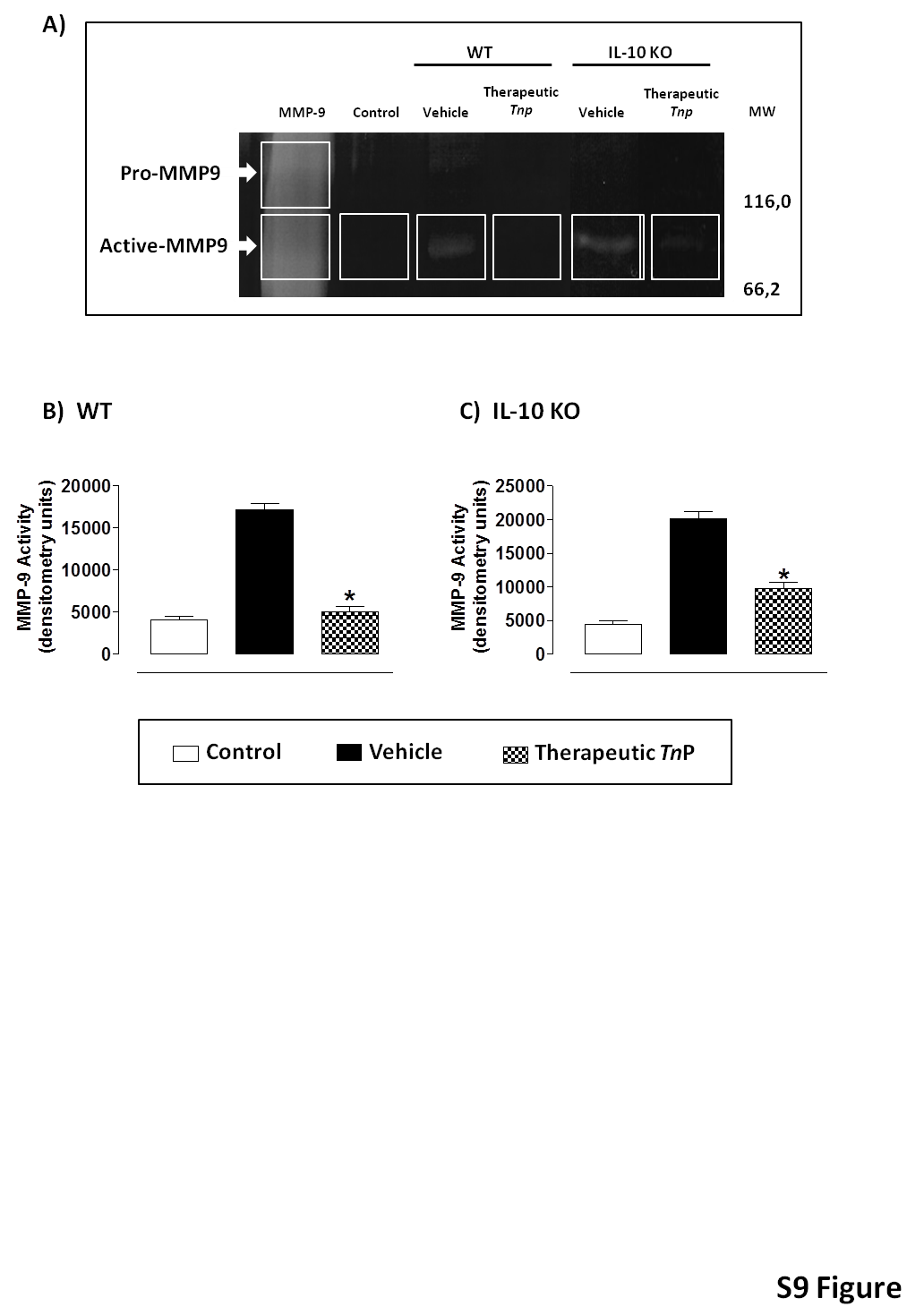

Supplement: S9 Fig — Gelatin gel zymography of pooled tissue homogenates of spinal cords at day 17 from WT or IL-10 KO EAE mice treated with vehicle or TnP and healthy mice (n = 5/group) shows active and pro-form of MMP-9 (A). Bar graphs (B and C) show densitometry quantification of representative gel zymography data. rMMP-9 was used as standard. Data represent mean ± SEM. *p < 0.05 compared with vehicle-treated EAE-mice. (TIF) [file pone.0171796.s009.tif]

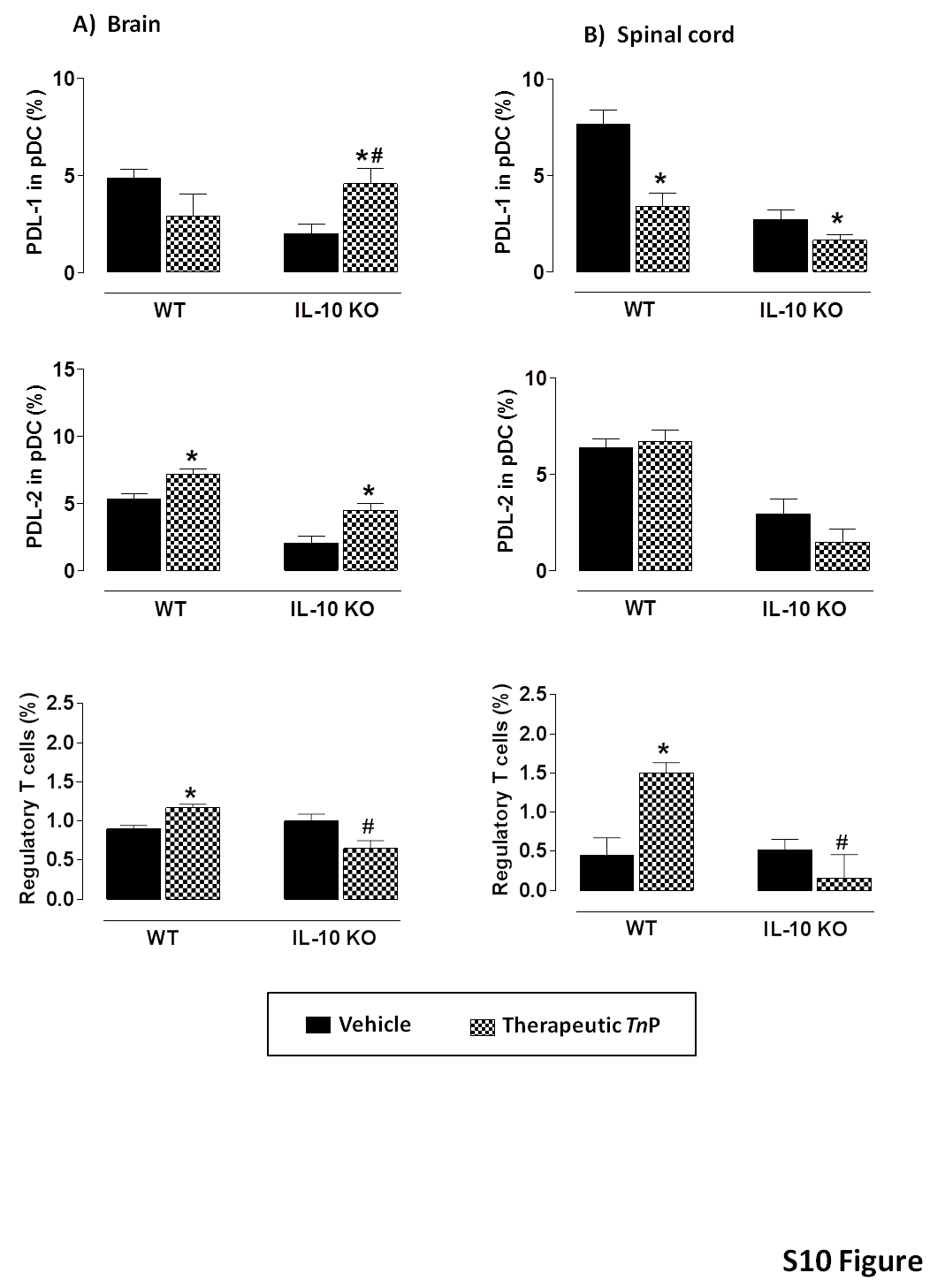

Supplement: S10 Fig — At the peak of disease, CNS-infiltrating leukocytes were isolated from pooled brain (A) and spinal cord (B) homogenates of WT or IL-10 KO EAE mice treated with vehicle or TnP (n = 5/group), and the percentages of PDL-1- and PDL-2-positive pDC (CD11c+CD45R/B220low) or the percentage of FOXP3-positive CD4+CD25+ Treg were evaluated after acquisition of 50,000 events. Data represent mean ± SEM. *p < 0.05 compared with vehicle-treated EAE-mice and # p < 0.05 compared with WT TnP-treated EAE-mice. (TIF) [file pone.0171796.s010.tif]

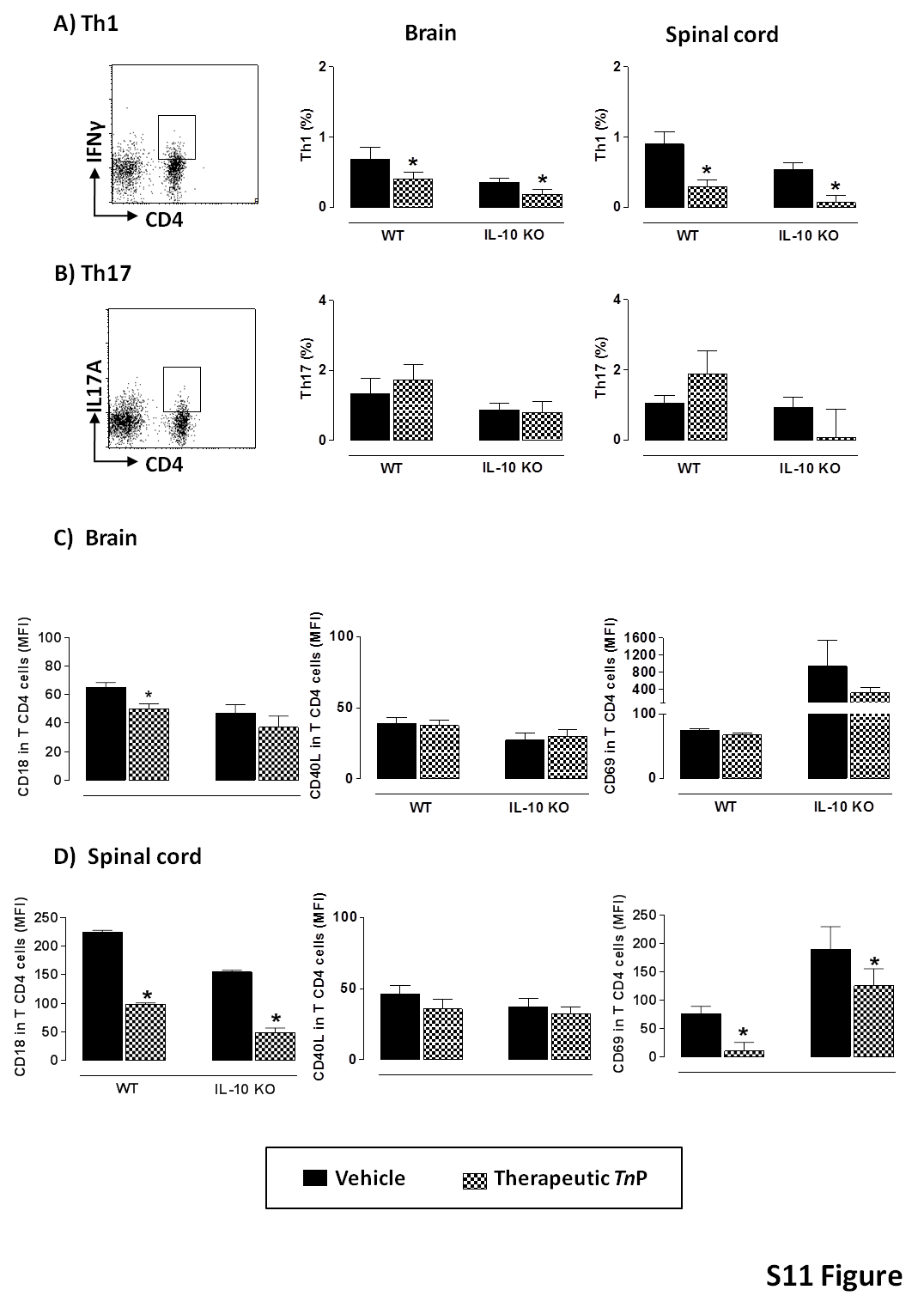

Supplement: S11 Fig — At day 17, CNS-infiltrating leukocytes were isolated from pooled brain and spinal cord homogenates of WT or IL-10 KO EAE mice treated with vehicle or TnP (n = 5/group). Percentages of Th1 (A) and Th17 (B) cells are shown in the indicated gates after acquisition of 50,000 events. CNS-infiltrating CD4 T lymphocytes were evaluated by the expression (MFI) of CD18, CD40L, and CD69 in brain (C) or in spinal cords (D). Values in the bar graphs are the mean ± SEM. *p < 0.05 compared with vehicle-treated EAE-mice. (TIF) [file pone.0171796.s011.tif]
